# Low-Dose Ozone as a Eustress Inducer: Experimental Evidence of the Molecular Mechanisms Accounting for Its Therapeutic Action

**DOI:** 10.3390/ijms252312657

**Published:** 2024-11-25

**Authors:** Manuela Malatesta, Gabriele Tabaracci, Carlo Pellicciari

**Affiliations:** 1 Department of Neurosciences, Biomedicine and Movement Sciences, University of Verona, 37134 Verona, Italy; 2 San Rocco Clinic, 25018 Montichiari, Italy; tabaracci@sanrocco.net; 3 Department of Biology and Biotechnology, University of Pavia, 27100 Pavia, Italy; carlo.pellicciari@unipv.it

**Keywords:** ozone, oxidative stress, Nrf2, inflammation, cytokines, apoptosis, autophagy, pain, cancer, tissue regeneration

## Abstract

Ozone (O_3_) is an unstable, highly oxidative gas that rapidly decomposes into oxygen. The therapeutic use of O_3_ dates back to the beginning of 20th century and is currently based on the application of low doses, inducing moderate oxidative stress that stimulates the antioxidant cellular defences without causing cell damage. In recent decades, experimental investigations allowed the establishment of some basic mechanisms accounting for the therapeutic effects of eustress-inducing low-dose O_3_. In this review, special attention was given to the impact of O_3_ administration on the cell oxidant–antioxidant status, O_3_ anti-inflammatory and analgesic properties, efficacy in improving tissue regeneration, and potential anticancer action. Low O_3_ concentrations proved to drive the cell antioxidant response mainly by activating nuclear factor erythroid 2-related factor 2. The anti-inflammatory effect relies on the downregulation of pro-inflammatory factors and the modulation of cytokine secretion. The painkilling action is related to anti-inflammatory processes, inhibition of apoptosis and autophagy, and modulation of pain receptors. The regenerative potential depends on antioxidant, anti-inflammatory, anti-apoptotic, and pro-proliferative capabilities, as well as fibroblast activation. Finally, the anticancer potential is based on oxidant and anti-inflammatory properties, as well as the inhibition of cell proliferation, invasion, and migration and the induction of apoptosis.

## 1. Introduction

Ozone (O_3_) occurs in nature as a chemical compound made of three oxygen atoms. It is highly unstable and decomposes into oxygen molecules with a half-life of 40 min at 20 °C. Its relative concentration in the atmosphere is very low (approximately 6.5 × 10^−7^ of the whole mass), with a mean concentration of 1 mg/m^3^. O_3_ is present in the entire atmosphere, from the ground up to approximately 50 km, but is unevenly distributed as its concentration increases with the distance from the Earth’s surface and reaches a maximum at altitudes of 20 to 25 km. Here, a millimetre-thick O_3_ layer absorbs most of the sun’s ultraviolet radiation, thus being responsible for the stratosphere’s thermal structure, while exerting a crucial protective role for all living organisms on Earth [1].

O_3_ was first detected in 1785 by the Dutch physician, chemist, and plant physiologist, Martinus van Marum, who noticed the typical smell acquired by air that had been passed by an electric spark [2]; he attributed the smell to the electricity itself, so it became known as “the odour of electricity” [3]. It was only in 1839 that the German–Swiss chemist Christian Friedrich Schönbein discovered O_3_ as a distinct chemical substance and gave it its name from the Greek word ὄζειν (ozein, meaning “to smell”) [4]. Schönbein demonstrated that O_3_ is a powerful oxidizing agent that reacts with inorganic [5] and organic compounds [6]. After it was demonstrated that O_3_ is an allotropic form of oxygen [7,8], the correct molecular formula for O_3_ was finally due to Andrews and Tait [9].

## 2. A Brief History of Medical Ozone

Contrary to its beneficial effects when in the stratosphere, O_3_ exerts a quite opposite role in the troposphere because of its highly oxidative power, which may be harmful to health, particularly breath. This was already reported by Schönbein [10], who observed that O_3_ at high concentrations affected breathing and caused chest pains and irritation of the mucous membranes in both humans and animals (mice and rabbits).

In 1857, von Siemens constructed the first technical unit for O_3_ production [11]. Since then, the use of such devices allowed better comprehension of the effects of O_3_ on living organisms. Fox [12] discovered that O_3_ is able to eliminate microorganisms, and this disinfectant action was later confirmed by Kellogg [13].

In 1896, Nikola Tesla was issued a patent for an O_3_ generator (US Patent 568,177; 22 September 1896); after the Tesla Ozone Company had been established in 1900, O_3_ generators were made available to medical doctors for practical use, and the company also produced and sold a medical gel made of ozonated olive oil.

The way was paved for the therapeutic application of O_3_, and the efficacy of O_3_ in treating different pathological conditions (e.g., severely infected wounds, periodontitis, proctitis, and chronic colitis) was practically established thanks to the pioneering works by physicians such as E. Payr, E. Fish, P. Aubourg, and H. Wolff. In 1929, Thauerkauf and Luth [14] opened an institute for oxygen therapy in Berlin, which was the first in the world. However, for therapeutic use of O_3_ to become increasingly popular and more widely applied as an adjunctive medical treatment [15,16,17,18,19,20,21], it was necessary to wait until the 1950s (with the advent of O_3_-resistant plastics) and the 1990s (when O_3_ generators able to precisely measure gas concentrations in real time were made commercially available).

Nowadays, medical O_3_ is commonly applied as a gaseous O_2_-O_3_ mixture, as ozonated oil or ozonated water, and may be administered both systemically (via bloodstream, rectal insufflation, or orally) and locally (via injection or topical application). Regardless of the formulation and the administration route, modern therapeutic applications of O_3_ are based on the low-dose concept according to Viebahn-Hänsler et al. [22,23]. In fact, experimental findings (detailed in the following sections) demonstrated that beneficial effects occur only within a range of low O_3_ concentrations (generally from 10 to 50 µg O_3_/mL O_2_); under these conditions, moderate oxidative stress is induced, which stimulates antioxidant and anti-inflammatory responses without causing cell damage [24]. This is consistent with the evidence that oxidative stress is not only responsible for deleterious effects (so-called distress) but may also act as a favourable messenger activating defensive pathways. This latter condition is defined as eustress [25,26], which occurs when stressors induce an adaptive, protective response through the alteration or modulation of gene expression. In fact, low levels of oxidative stress were found to enhance the defence capability of cells and tissues by increasing the expression of antioxidant compounds [27,28]. The concept of eustress has also been applied to medical low-dose O_3_ and is recognised as a master mechanism of action at the root of cascades of biological events leading to therapeutic effects [29,30].

## 3. Methods and Scope of the Present Review

Information on studies investigating the molecular pathways accounting for the beneficial effects of medical O_3_ administration was obtained from the PubMed database. Only articles in English dealing with medical O_3_ (i.e., used for therapeutic purposes) were selected. No studies using high O_3_ concentrations were considered; instead, only low doses were considered because the point of the present review is to illustrate the eustress-driven biological effects of O_3_. Moreover, no clinical studies were considered, and only experimental investigations performed in in vitro, ex vivo (animals and humans), and in vivo (animals) systems were selected. In fact, while clinical observational studies are irreplaceable for assessing the therapeutic efficacy of O_3_, only experimental studies allow understanding and mechanistic explanations of the cellular and molecular processes due to the use of strictly controlled test conditions and refined analytical techniques, which can hardly be applied to patients. For Section 4, Section 5, Section 6, Section 7 and Section 8, only research articles were considered; articles describing molecular mechanisms are reported in detail, while articles reporting observational results are cited in a limited number. For Section 1, Section 2, and Section 9, review articles and book chapters were also used.

The aim of the present review is to provide an overview of the current knowledge on the cellular and molecular mechanisms responsible for the effects of low O_3_ doses on some physiological or pathological conditions that have extensively been investigated over the last three decades; namely, the impact of O_3_ administration on cell oxidant–antioxidant status, anti-inflammatory and analgesic properties, efficacy in improving tissue regeneration, and potential anticancer action.

## 4. Ozone and Oxidative Stress

Key information on the molecular mechanisms responsible for the impact of low O_3_ concentrations on oxidant–antioxidant status was obtained from in vitro studies on human blood.

Bocci et al. [31,32,33] demonstrated that O_3_ administered ex vivo to whole human blood generated reactive oxygen species (ROS), with hydrogen peroxide (H_2_O_2_), lipid oxidation products, and oxidised thiol groups being mostly present in albumin. Moreover, O_3_ reduced glutathione (GSH) while concomitantly increasing its oxidised form (GSSG) in erythrocytes. All these modifications were transient, and normal values were restored in a short time (from 1 to 20 min), demonstrating that blood contains antioxidant factors that effectively quench the formation of ROS and can regenerate oxidised antioxidants. These events were observed in the concentration range of 10 to 80 µg O_3_/mL O_2_; lower concentrations were ineffective because of the instant O_3_ quenching by blood antioxidants, while higher concentrations were harmful because they overwhelm the blood antioxidant capacity. The authors concluded that, at appropriate doses, O_3_ may induce safe oxidative reactions generating several messengers (including H_2_O_2_ and 4-hydroxynonenal (4-HNE)), which can exert important biological activities without damaging blood components.

Subsequently, a metabolomic study on human blood treated ex vivo with O_3_ revealed an increase in formate, allantoine, acetoacetate, and acetate and a decrease in pyruvate. These modifications were correlated to the direct oxidant activity of O_3_ on plasma components such as pyruvate, carbohydrates, and uric acid [34]. A further metabolomic study on ex vivo-treated human blood demonstrated that O_3_ induced changes in plasma antioxidants (bilirubin, biliverdin, pyroglutamic acid, and dihydroxyvitamin D3), an increase in lactic and pyruvic acid (indicating an increased rate of glycolysis), and a rise in lipid oxidation products, lysophospholipids, and oxidised forms of phosphatidylcholines (as markers of oxidative stress) [35]. Recently, metabolomic analyses added further information on the molecular effects induced ex vivo by O_3_ in blood by revealing changes in metabolites of amino acids (e.g., N-acetyl-L-alanine, 5-hydroxytryptophan, L-glutamic acid, maleic acid), carbohydrates (e.g., D-glucose, glutaric acid, malic acid, aceturic acid), lipids (e.g., behenic and stearic acid, pimelic acid, oleic and linoleic acid, erythrono-1,4-lactone, arachidic acid, valeric acid), and nucleotides (e.g., niacinamide, glyceric acid, uridine), which are mostly involved in antioxidant and anti-inflammatory pathways, as well as energy production [36].

In vitro studies using cell lines allowed deepening of the knowledge of the antioxidant response induced by O_3_ treatment. By using a human T lymphoma cell line, it was demonstrated that O_3_ induces a dose-dependent increase in the activity of superoxide dismutase (SOD), glutathione peroxidase (GPx), and glutathione reductase, while glucose-6-phosphate dehydrogenase (G6PDH) showed a peak following exposure to a concentration of 12 µg O_3_/mL O_2_ [37]. By treating in vitro human vascular endothelial cells with ozonated human plasma, Bocci et al. [38] reported upregulation of haeme oxygenase-1 (HO-1, also known as HMOX1, which plays a key role in antioxidant defence) and Heat Shock Protein 70 (Hsp-70, which is involved in cell protection from various stresses), which was accompanied by a rise in nitric oxide (NO, known to modulate HO-1 activity). Accordingly, pathway analysis of all the genes modulated by different O_3_ concentrations showed that in human SH-SY5Y neuronal cells, the only common pathway was the signalling associated with HO-1, which indicates that this enzyme plays a primary role in the cellular response to O_3_ treatment [39].

Extensive demonstrations of the O_3_-driven antioxidant response were also obtained in vivo. León et al. [40] demonstrated that O_3_ rectal insufflation prevented rats from the free-radical-mediated hepatic injury induced by carbon tetrachloride poisoning by maintaining several biochemical parameters at control levels, i.e., transaminase, cholinesterase, SOD, catalase (CAT), phospholipase A, calcium-dependent adenosine triphosphatase (ATPase), GSH, and G6PDH, and lipid peroxidation. In the following decades, many experimental studies confirmed and extended the notion that O_3_ administration is able to protect several organs from the damage caused by various oxidizing agents or in experimentally induced pathological conditions; such a protection depends on the enhanced activity of antioxidant enzymes not only in the liver (e.g., [41,42,43,44]) but also in the kidney (e.g., [45,46,47,48]), heart (e.g., [49]), urinary bladder (e.g., [50]), testis (e.g., [51,52,53]), intestine (e.g., [43,54,55]), stomach (e.g., [56]), oesophagus (e.g., [57]), pancreas (e.g., [58,59]), lung (e.g., [42,60]), nervous tissue (e.g., [61]), and skin (e.g., [62]).

Similarly, pre- and post-treatment with O_3_ proved to enhance the antioxidant enzyme activity in several organs undergoing ischaemia–reperfusion, thus limiting oxidative stress injury in the liver (e.g., [63,64,65]), kidney (e.g., [66,67]), intestine (e.g., [68,69]), heart (e.g., [65,70]), muscle (e.g., [71]), bone (e.g., [72]), nervous tissue (e.g., [73,74]), cochlea (e.g., [75]), retina (e.g., [76]), ovary (e.g., [77,78]), and testis (e.g., [79]).

Ageing is characterised by chronic oxidative stress, and O_3_ was found to alleviate the age-associated imbalance of the redox state in various experimental models. O_3_ administration for twelve months to rats proved to ameliorate some age-related alterations in the cerebral cortex. In detail, after O_3_ treatment, the GSH content, the adenosine triphosphate (ATP):adenosine diphosphate (ADP) ratio, and mitochondrial SOD and complex IV (cytochrome-c oxidase) activities were normalised, the glutathione redox index and the activities of mitochondrial complex I (nicotinamide adenine dinucleotide-ubiquinone oxidoreductase) and NO synthase (mtNOS) were improved, and the increases in the levels of malondialdehyde (MDA, an end product of membrane lipid peroxidation) and mitochondrial protein carbonyl (PCO) were limited [80]. Long-term pre-ageing administration of O_3_ to rats also proved to counteract age-related oxidative stress in the liver and kidneys by reducing MDA and PCO levels, restoring normal levels of GSH and GPx, and decreasing lipofuscin deposition. Similar, though less pronounced, effects were obtained by treating aged rats with O_3_ for one month [81]. In the heart and hippocampus of aged rats treated for twelve months with O_3_, decreases were observed in MDA, PCO, and GSH levels, while GPx activity was normalised; in addition, O_3_ reversed age-associated decreases in ATP and the ATP:ADP ratio in both tissues, restored the attenuated Na^+^, K^+^-ATPase activity in the hippocampus, and the decreased cytosolic Ca^2+^ levels in the heart [82]. In aged rats, two-month-long O_3_ treatment was able to reduce oxidative stress and apoptosis, as demonstrated by downregulation of inducible NOS (iNOS) and caspase-3 (a pro-apoptotic factor), respectively. In parallel, O_3_-treated old rats showed increased levels of Mab2 (a marker of neurogenesis) according to an increased number of Purkinje cells and decreased levels of the astrocyte marker, glial fibrillary acidic protein [83].

In vitro and in vivo studies demonstrated the key role of the nuclear factor erythroid 2-related factor 2 (Nrf2) in the antioxidant response generated by low O_3_ concentrations. By treating a human endothelial cell line with ozonated human serum, Pecorelli et al. [84] observed increases in the levels of Nrf2 protein and Kelch-like ECH associated protein 1 (Keap1) in the nucleus and concomitant decreases in their contents in the cytoplasm. Importantly, these effects were only detected at concentrations ranging from 20 to 80 µg O_3_/mL O_2_, whereas no effect was observed at higher O_3_ doses. When the cells were treated with 4-HNE or H_2_O_2_ (i.e., with molecules that are generated by the reaction of O_3_ with serum components), nuclear translocation of Nrf2 took place, as it occurs after exposure to ozonated serum, which suggests that these messenger molecules are the main responsible entities for Nrf2 activation. Moreover, ozonated serum also induced increases in the protein levels of HO-1 and nicotinamide adenine dinucleotide phosphate quinone oxidoreductase 1 independent from extracellular signal-regulated kinase (ERK) 1/2 and p38 (both belonging to the mitogen-activated protein kinase (MAPK) family). Accordingly, activation of Nrf2 pathway and promotion of antioxidant enzymatic systems was observed in healthy humans submitted to systemic administration of low-dose gaseous O_3_ [85]. A mechanistic demonstration for these events was provided by Galiè al. [86]; by applying transfection methods to an in vitro cell model, low O_3_ concentrations were found to promote the nuclear translocation of Nrf2 to active transcription sites, massively inducing the expression of antioxidant response element (ARE)-driven genes, which encode detoxification enzymes and cytoprotective proteins, including HO-1. Moreover, the expression of the Nrf2-specific inhibitor Keap1 was found to revert O_3_-induced ARE activation, thus unequivocally demonstrating the role of the Nrf2 pathway in the O_3_-driven antioxidant response.

In a rat model of streptozotocin-induced pancreatic damage, systemic O_3_ treatment improved pancreas functionality by increasing Nrf2 and glutathione-s-transferase and reducing the levels of 4-HNE and poly(ADP-ribose) polymerase-1 (PARP-1, which is involved in chromatin remodelling and apoptosis), thus demonstrating that both the antioxidant and anti-apoptotic processes were efficiently activated [87]. Ding et al. [88] established an in vivo mouse model of ischaemia–reperfusion injury and an in vitro cardiomyocyte model of hypoxia-reoxygenation injury to investigate the mechanism accounting for the protective action of O_3_ on the myocardium. They demonstrated that O_3_ not only promoted nuclear translocation of Nrf2 but also inhibited ferroptosis (a form of oxidative cell death) and induced the expression of the antioxidant factors solute carrier family 7 member 11 (Slc7a11) and GPx4. Since Nrf2 gene silencing reversed the protective effects of O_3_, it was evident that Nrf2 activation was the main responsible event for such a response. By using a rat model of kidney transplantation, Qiu et al. [89] found that the expression of Nrf2 and HO-1 increased in O_3_-treated animals, suggesting that the mechanism by which O_3_ alleviates oxidative stress injury in renal transplantation may be related to activation of the Nrf2/HO-1 signalling pathways and inhibition of apoptosis in tubular epithelial cells. In a rat model of chronic kidney disease, O_3_ attenuated tubulointerstitial injury, restored Nrf2 activation, and inhibited the pathway of the nuclear factor kappa-light-chain-enhancer of activated B cells (NF-κB, which plays a key role in the cell response to harmful stimuli and in regulation of the immune response) [90]; O_3_-driven modulation of Nrf2 and NF-κB also resulted in upregulation of antioxidation enzymes (SOD, CAT, and GSH) and downregulation of oxidation products (MDA and PCO) and inflammatory cytokines (interleukin (IL)-1β, IL-6, tumour necrosis factor (TNF)-α, and intercellular adhesion molecule-1). In a rat model of lung ischaemia–reperfusion injury, O_3_ was found to drastically reduce oxidative stress, inflammation, and apoptosis [91]. The molecular mechanisms underlying this protective effect included increased expression of Nrf2 and decreased levels of NOD-like receptor family pyrin domain containing 3 (NLRP3), apoptosis-associated speck-like protein containing a caspase activation and recruitment domain (ASC), procaspase-1, caspase-1, and IL-1β. Finally, using a rat model of cerebral ischaemia–reperfusion injury, Zhu et al. [92] demonstrated that O_3_ can limit neuronal damage by reducing ferroptosis through the Nrf2/SLC7A11/GPx4 pathway, particularly by increasing the nuclear translocation of Nrf2 and the expression of SLC7A11 and GPx4.

Studies on different experimental models submitted to oxidative stress have demonstrated that O_3_ treatment alleviates oxidative damage not only by increasing the activity of antioxidant enzymes through Nrf2 but also through other molecular pathways.

In a rat model of experimentally induced seizures, O_3_ treatment was found to mitigate seizure severity and improve survival; besides increasing lipid peroxidation and reducing SOD activity, O_3_ increased adenosine availability to activate A1 adenosine receptors, which is known to have antiepileptic and neuroprotective functions [93]. Consistently, in a rat model of liver ischaemia–reperfusion, O_3_ was found to limit increases in NO, NF-κB, TNF-α, and Hsp-70 in hepatic tissue while activating A1 adenosine receptors [94]. This suggested that O_3_ may protect the liver from oxidative injury through activation of A1 adenosine receptors, promoting cellular signalling for preservation of the cellular redox balance. In kidneys undergoing ischaemia–reperfusion, O_3_ proved to increase NO and NOS expression and to inhibit the depletion of SOD, GSH, and GPx in renal tissue [95,96], suggesting that the antioxidant protective effect of O_3_ is closely related to NO production following increases in its endothelial NOS and iNOS expression. On the other hand, in a rat model of skeletal muscle ischaemia–reperfusion, administration of O_3_ decreased the muscle contents of NO, iNOS, MDA, and IL-1β, while the levels of SOD and GPx increased, thus indicating that O_3_ may counteract both oxidative and nitrosative stress [97]. An in vivo and in vitro study of kidney ischaemia–reperfusion demonstrated that O_3_ can also inhibit the activation of MAPK, resulting in protection against apoptosis and oxidative stress [98]. Moreover, O_3_ was found to attenuate kidney tubulointerstitial fibrosis by downregulating α-smooth muscle actin (α-SMA, which is involved in myofibroblastic differentiation), transforming growth factor β1 (TGF-β1, which is involved in cell proliferation, differentiation, and apoptosis), and phospho-small mother against decapentaplegic (SMAD) 2 protein (a main signal transducer for receptors of the TGF-β superfamily) [99]. O_3_-induced inhibition of autophagy was observed in a rat model of cerebral ischaemia, where O_3_ administration decreased the expression of Beclin-1 (involved in the regulation of autophagy and cell death) and microtubule-associated protein 1A/1B-light chain 3 (LC3) (involved in autophagy) and increased the expression of p62 (known to decrease when autophagy is induced) [100]. This is consistent with observations in an in vitro model of the myocardium undergoing oxygen-glucose deprivation/reperfusion: here, O_3_ pre-treatment prevented decreases in the ratio of B-cell lymphoma 2 (Bcl-2)/Bcl2-associated X protein (Bax) and cleaved caspase-3 (which plays key roles in apoptosis regulation and execution) while reducing the LC3-II/LC3-I ratio and the levels of Beclin-1 and autophagy protein 5 (Atg5, which is essential for autophagic vesicle formation) [101]. These findings suggested that O_3_ is able to counteract the oxidative stress induced by oxygen–glucose deprivation/reperfusion through inhibition of the autophagy pathway. Finally, in an ex vivo rabbit model of heart ischaemia–reperfusion, O_3_ significantly increased the expression of IL-10 and hypoxia inducible factor-1α (HIF-1α, a transcription factor that regulates the cell response to hypoxia), decreased the expression of IL-6, and reduced the concentrations of creatine kinase-MB and troponin T (which are markers of myocardium damage) [102]. After treatment with a specific inhibitor of HIF-1α, the protective action of O_3_ was reversed completely, so the authors hypothesised that the observed effects of O_3_ rely on the upregulation of HIF-1α, which is vital for attenuating mitochondrial damage, maintaining normal mitochondrial respiratory energy metabolism, and reducing the generation of mitochondrial ROS.

Since mitochondria play a central role in ROS generation and in the maintenance of the cell oxidant/antioxidant balance, some experimental studies focussed on the effects of O_3_ on mitochondrial function. In a rat model of endotoxaemic shock characterised by hypoxia, O_3_ treatment partially restored the decreased mitochondrial function in the liver and heart by increasing the activity of the respiratory enzymes succinate dehydrogenase and ATPase and by decreasing lactate dehydrogenase (LDH) [103]. In a rat model of noise-induced hearing loss, Nasezadeh et al. [104] investigated the protective effect of O_3_ on mitochondria from the brain and cochlea. O_3_ treatment prevented the decrease in GSH and the increase in GSSG induced by noise, enhanced the activity of CAT, SOD, and GPx, and decreased the MDA level. In isolated mitochondria from O_3_-treated animals, ROS production and cytochrome c release decreased, membrane potential and ATP levels increased, and mitochondrial swelling induced by noise was reverted. In an in vitro study [105], treatment of mouse myoblasts with different concentrations of O_3_ did not alter mitochondrial structure and reduced ROS production at the lowest dose, whereas a higher dose induced mitochondrial swelling and increased ROS production; in addition, O_3_ proved to modulate the association of Nrf2 with the outer mitochondrial membrane. Oliveira et al. [106] investigated the response of hepatic mitochondria to O_3_ treatment in mice: under the tested experimental conditions, O_3_ reduced mitochondrial respiration by inhibiting the activity of electron transport chain complexes I and II/III. In addition, the activity of SOD and GPx increased, while the GSH content decreased. Since ATP levels remained unchanged, the authors hypothesised that such mitochondrial inhibition prevented mitochondrial ROS production without affecting the energy charge.

**Figure 1:**
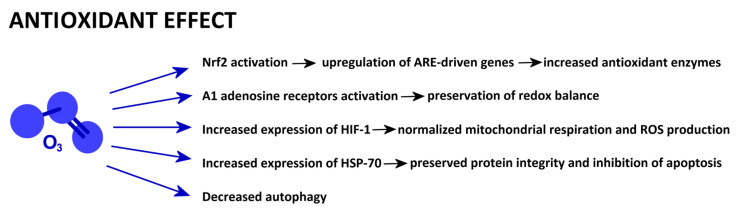
Low-dose O_3_-driven cell mechanisms involved in antioxidant properties.

A schematic presentation of the mechanisms reported in this section is given in Figure 1.

## 5. Ozone and Inflammation

As reported in the previous section, O_3_ is able to exert anti-inflammatory effects. Therefore, many studies have been performed to investigate changes in cytokines, ILs, and other factors involved in inflammation following treatment with low O_3_ concentrations (as gas or ozonated water/oil).

The first observations were made on blood. After treatment with O_3_ ex vivo, human whole blood showed increases in IL-1β, IL-2, IL-6, granulocyte-macrophage colony-stimulating factor, interferon (IFN)-β, IFN-γ, TNF-α [31,107], TGF-β1 [108], and IL-8 [109]. Human leukocytes treated with O_3_ released IFN-γ [110] and TNF-α [111] and inhibited the release of IL-4, IL-6, and IL-10 [112]. After O_3_ treatment, human platelets were found to release platelet-derived growth factor (PDGF), TGF-β1, and IL-8 [113]. In addition, treatment with O_3_ of human platelet-rich plasma (PRP) proved to increase the platelet release of IL-2, IFN-α, epidermal growth factor, fibroblast growth factor 2 (FGF-2, a mitogenic factor involved in cell growth, morphogenesis, and tissue repair secreted by inflammatory cells, endothelial cells, and myofibroblasts), and vascular endothelial growth factor (VEGF, which plays a key role in vasculogenesis and angiogenesis) [114]. Blood ozonation also induced a transient increase in TNF-α in rabbits in vivo [115]. By treating in vitro-cultured human endothelial cells with ozonated human serum, the release of IL-8 was stimulated, while E-selectin (a protein expressed by inflamed endothelial cells) was inhibited [116].

Besides blood cells, O_3_ demonstrated anti-inflammatory effects in vitro on a human colon cancer cell line by inducing the expression of TNF-α, matrix metalloproteinase (MMP)-2, and MMP-9 (both of which are involved in the breakdown of extracellular matrix and activation/cleavage of cytokines such as TGF-β and IL-1β) while reducing the production of IL-1β, IL-6, and IL-8 [117].

O_3_ also proved to significantly reduce pro-inflammatory cytokines (e.g., IL-1β, IL-2, IL-6, IL-12, IFN-γ, TNF-α) and increase anti-inflammatory ILs (e.g., IL-10) in vivo when administered to animal models of pathological conditions or ischaemia–reperfusion, as reported in the liver (e.g., [118,119]), kidney (e.g., [120,121,122]), joints (e.g., [123,124]), lung (e.g., [125]), intestine (e.g., [126]), uterus (e.g., [127,128]), mammary glands (e.g., [129]), eye (e.g., [130,131]), skin (e.g., [132]), and brain (e.g., [133]).

Some in vitro and in vivo studies therefore focused on the mechanisms and molecular pathways involved in the anti-inflammatory effect of O_3_. In a rat model of adenine-induced chronic kidney disease, O_3_ was found to limit inflammation injury in the tubulointerstitium. O_3_ suppressed the expression of toll-like receptor 4 (TLR4) and phosphorylated NF-κB p65 (p-NF-κB p65), which both stimulate inflammation cascades when excessively activated. In addition, TLR4 levels positively correlated with monocyte chemoattractant protein-1, TNF-α, IL-1β, and IL-6, which were found to decrease after O_3_ treatment. These findings suggested that O_3_ alleviates tubulointerstitial inflammation injury through modulation of TLR4 [134]. Similarly, in a rat model of cerebral ischaemia, O_3_ inactivated the NF-κB signalling pathway by decreasing the protein levels of TLR4, phosphorylated inhibitor of NF-κB kinase subunits α and β (p-IKBα and p-IKKβ), and phosphorylated p65 (p-p65), thus reducing inflammation [100]. O_3_ was also found to suppress activation of TLR4/NF-κB signalling in a rat heart myoblast line treated with doxorubicin, thus leading to reductions in IL-1β, IL-6, TNF-α, MMP-2, and MMP-9 expression [135].

O_3_ induced an anti-inflammatory response in a human melanoma cell line by reducing activation of p65/NF-κB, which is involved in the inflammatory signalling pathway and is responsible for the genesis of many pro-inflammatory ILs. Consequently, there was a decrease in the production of IL-1, IL-6, IL-8, IL-9, IL-17, IL-19, TNF-α, TGF-β, VEGF, MMP-2, and MMP-9 [136]. Accordingly, O_3_ mitigated the cytotoxic and pro-inflammatory effects of doxorubicin on cultured human skin fibroblast cells and human foetal cardiomyocytes by reducing the expression of the pro-inflammatory factors p65/NF-κB and leukotriene B4. Moreover, O_3_ enhanced Nrf2 expression; as a result, the production of IL-1, IL-8, IL-6, TNF-α, MMP-2, and MMP-9 induced by doxorubicin was markedly reduced [137]. A reduction in the expression of p65/NF-κB following O_3_ treatment has also been reported in the kidney of a rat model of renal transplantation, which was accompanied by a decrease in high-mobility group box-1 (HMGB1) protein, a chromatin-associated regulator of nuclear transcription involved in inflammatory events. Accordingly, the serum levels of IL-6, IL-18, and cyclooxygenase (COX)-2 (responsible for the production of immune-suppressive prostaglandins) were lowered in transplanted rats treated with O_3_ [67]. Reduced levels of NF-κB p65 were found in a rat endotoxic shock model treated with O_3_, where morphological signs of decreased inflammation were observed in hepatic tissue [138]. NF-κB modulation in the O_3_-driven anti-inflammatory action has been reported in an in vitro model of human lung alveolar cell injury induced by H_2_O_2_ oxidative stress [139]. O_3_ was in fact found to suppress the expression of NF-κB, TNF-α and Bax genes, thus reducing inflammation and cell death. In addition, O_3_ significantly increased mRNA levels of SOD, CAT, and GPx, thus improving cell antioxidant defence.

Besides suppression of NF-κB, Nrf2 stimulation was also found to play a role in the anti-inflammatory action of O_3_.

In lipopolysaccharide (LPS)-stimulated human colonic epithelial and monocytic cell lines, ozonated oil increased the expression of Nrf2 and reduced various pro-inflammatory factors, including IL-1β, TNF-α, NOS2, and MMP-2 [140]. In vitro studies on human fibroblast [141] and microglia [142] cell lines revealed that O_3_ differentially modulates the release of IL-6, TNF-α, and TGF-β1 in LPS-activated and non-activated cells. This effect was in turn related to the differential O_3_-driven activation of Nrf2 in activated and non-activated cells. In rats experiencing lung ischaemia–reperfusion, O_3_ increased the expression of Nrf2 and decreased the levels of the NLRP3 inflammasome, thus reducing oxidative stress and inflammation. These processes were accompanied by anti-apoptotic effects based on reductions in ASC, procaspase-1, caspase-1, and IL-1β [91].

Yu et al. [143] further investigated the role of the NLRP3 inflammasome in the reno-protective effect of O_3_ in chronic kidney disease. Using nephrectomised rats, the authors observed that IL-1β levels positively correlated with decreased expression of NLRP3 following O_3_ treatment. It was therefore hypothesised that O_3_ exerts anti-inflammatory activity through modulation of the NLRP3 inflammasome. Similarly, Wang et al. [144] investigated the effects of ozonated triglyceride (a component of ozonated oil) in a mouse model of sepsis and in vitro using primary peritoneal macrophages and a human acute monocytic-leukaemia cell line. They demonstrated that ozonated triglyceride suppressed activation of NLRP3 inflammasome, which explained the observed inhibition of caspase-1 cleavage and the reduced release of the inflammasome-related cytokines IL-1β and IL-18.

Other inflammation-related signalling pathways have been found to be modulated by O_3_.

Yan et al. [145] investigated the anti-inflammatory potential of O_3_ on sepsis-induced acute lung injury using a mouse in vivo model and primary cultures of mouse bone marrow cells. They found that O_3_ treatment induced significant decreases in the levels of IL-1β, MMP-9, and tissue factor (TF, which is involved in pro-inflammatory and pro-angiogenic pathways) in lung tissue and promoted the phagocytic function of macrophages by upregulating the expression of the Class A1 scavenger receptor (SR-A1) gene through 5′ adenosine monophosphate-activated protein kinase (AMPK) phosphorylation. SR-A1 is a pattern recognition receptor that functions in synergy with TLR4 and may promote anti-inflammatory responses. Based on these findings, the authors hypothesised that O_3_ alleviates septic lung injury via the AMPK/SR-A1 pathway, thus enhancing the phagocytosis of neutrophil extracellular traps (NETs) by macrophages.

Kim et al. [146] demonstrated that ozonated oil is able to reduce IL-1β and NO levels in an LPS-treated mouse macrophage cell line and the serum levels of IL-1β, TNF-α, NO, iNOS, and immunoglobulin E (IgE) in a mouse model of atopic dermatitis. Moreover, in mouse skin, the levels of IL-4, thymic stromal lymphopoietin (TSLP, a cytokine that regulates T-cell activity), phosphorylated signal transducer and activator of transcription 3 (STAT3, an inflammation-related transcriptional factor), and ERK were significantly reduced by ozonated oil treatment, while filaggrin (a keratin-bound protein in epithelial cells) levels increased. It is known that in keratinocytes of atopic dermatitis, IL-4 activates STAT3 to promote the transcription of TSLP, which in turn inhibits the expression of filaggrin by upregulating the STAT3/ERK pathway. Therefore, these findings suggested that the protective effects of ozonated oil on the skin barrier are mediated through inhibition of the IL-4/STAT3 signalling pathway. In addition, phosphorylated c-Jun N-terminal kinase (p-JNK, which is responsible for STAT3 phosphorylation), p38 (which belongs to the MAPK family), NF-κB, and Nrf2 were found to be decreased by ozonated oil. Since MAPK and NF-κB regulate the pro-inflammatory cytokines IL-1β and TNF-α, it was inferred that O_3_ exerts its anti-inflammatory effects by downregulating the phosphorylation of p38 and JNK and the expression of NF-κB. The reduction in Nrf2 expression was interpreted as a further effect of the anti-inflammatory properties of O_3_ since in atopic dermatitis, this protein is upregulated due the inflammatory response of skin.

**Figure 2:**
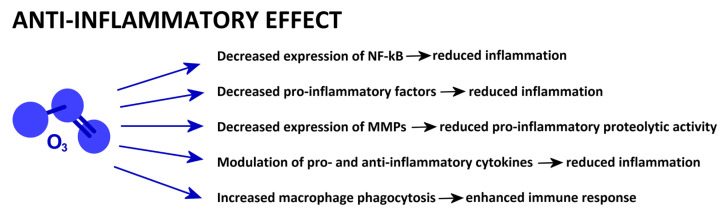
Low-dose O_3_-driven cell mechanisms involved in anti-inflammatory properties.

A schematic presentation of the mechanisms reported in this section is given in Figure 2.

## 6. Ozone and Pain

Experimental evidence for the biological mechanisms accounting for the analgesic properties of O_3_ is quite limited despite the extensive use of O_3_ as a painkiller in clinical practice and numerous retrospective clinical studies and case reports in the scientific literature.

In 2009, a preliminary study [147] demonstrated that a subcutaneous injection of O_3_ in a mouse neuropathic pain model (i.e., spared nerve injury of the sciatic nerve) decreased mechanical allodynia and reduced the expression of caspase-1, caspase-12, and caspase-8 genes, as well as the levels of IL-1β in the orbito-frontal cortex. These results suggested that O_3_ may modulate allodynia by regulating the expression of the genes that play a pivotal role in the onset and maintenance of this pain type.

A study was performed combining mathematical models of the intervertebral disc space, in vitro ozonolysis experiments using glycosaminoglycans from Chinese hamster ovary cells, and in vivo percutaneous intradiscal administration of O_3_ in Yucatan miniature pigs [148]. It was demonstrated that O_3_-fragmented disc proteoglycans, which reduce disc volume and compression on the nerve root, alleviated related pain. Moreover, O_3_ increased the levels of IL-1β, IL-6, IL-8, and TNF-α, likely contributing to improved symptomatology through their anti-inflammatory activity.

In order to elucidate the mechanisms underlying the painkilling potential of O_3_ in chronic radiculitis and mechanical allodynia after noncompressive lumbar disc herniation, Wang et al. [149] established a chronic radiculitis rat model: after intrathecal administration of O_3_, behavioural tests showed decreased pain, while molecular analyses demonstrated downregulation of spinal TNF-α, IL-1β, and IL-6, decreased mRNA and protein levels of spinal phosphodiesterase 2A (PDE2A, an enzyme catalysing the degradation of cyclic nucleotides and contributing to the processing of inflammatory pain) and NF-κB p65, and increased expression of cyclic guanosine monophosphate (cGMP) and cyclic adenosine monophosphate (cAMP). These findings suggested that O_3_ modulates pain through the PDE2A-cGMP/cAMP-NF-κB/p65 signalling pathway. The same research group demonstrated that O_3_ administered to a radiculoneuritis rat model decreased the expression of caspase-3, LC3, Beclin-1, PDE2A, and NF-κB p65, leading to inhibition of both autophagy and apoptosis in nerve root cells [150].

Lu et al. [151] used a rat model of chronic constriction injury of the sciatic nerve and a mouse macrophage cell line to demonstrate that peri-sciatic nerve injection of O_3_ was able to reduce neuropathic pain and normalise the phosphorylation of protein kinase C γ (expressed exclusively in brain spinal cord neurons and is involved in neuropathic pain development), N-methyl-D-aspartate receptor (a glutamate receptor), and ERK levels while reducing microglia activation. Importantly, all these events depended on the activation of AMPK, which was found to be phosphorylated during O_3_ treatment. Accordingly, Fan et al. [152] demonstrated that O_3_ is able to increase AMPK phosphorylation and the release of growth arrest-specific (Gas)-6 protein (involved in cell proliferation) to upregulate the proto-oncogene tyrosine-protein kinase MER (MerTK, which codes for transmembrane receptors that promote macrophage phagocytic activity) and cytokine signalling 3 (SOCS3, which regulates cytokine or hormone signalling to control inflammation homeostasis) and to reduce MMP-9 expression in a gouty mouse model and a mouse macrophage cell line. The authors concluded that the reduction in inflammation and pain takes place through activation of AMPK, which in turn upregulates the Gas6/MerTK/SOCS3 signalling pathway. Upregulation of the AMPK–SOCS3 axis induced by O_3_ treatment was also found to be responsible for the alleviation of neuropathic pain in a mouse oxaliplatin chemotherapy-induced peripheral neuropathy model [153]. In detail, O_3_ activated AMPK and induced SOCS3 expression, which decreased TF, c-Fos (involved in many functions concerning cell proliferation, differentiation, and survival) and calcitonin gene-related peptide (a potent vasodilator implicated in pain pathways) and inhibited microglial activation.

The mechanisms accounting for the pain-alleviating effect of O_3_ were investigated in a chronic constriction injury model of the sciatic nerve in rats. Behavioural test proved that intrathecal injections of O_3_ were able to lessen neuropathic pain by drastically reducing the expression of spinal glutamate receptor 6 (GluR6), IL-1β, IL-6, TNF-α, and NF-κB/p65, thus indicating a key role of GluR6 in the mitigation of neuropathic pain through the GluR6-NF-κB/p65 signalling pathway [154].

By using a transcriptomic and metabolomic approach, Yang et al. [155] investigated the effect of O_3_ treatment in the brainstem and hypothalamus of a rat model of neuropathic pain. They reported significant modifications in the expression of various genes, the most prominent of which being dorsal column stenosis 1 (involved in neural development and the regulation of neuropathic pain) and allograft inflammatory factor 1-like (involved in inflammation and immune responses). The levels of several other metabolites, such as aconitic acid, L-glutamic acid, uridine diphosphate-glucose, and tyrosine, were also modified, suggesting O_3_-induced modulation of energetic pathways and the production of pain-related neurotransmitters.

Yue et al. [156] reported that overexpression of the small nucleolar RNA host gene 16 (Snhg16, a regulator of cell proliferation, migration, and apoptosis) in a mouse model of chronic constriction injury diminished the efficacy of O_3_ treatment for neuropathic pain by binding to microRNA (miR)-719 and increasing the expression of sodium voltage-gated channel alpha subunit 1 (SCN1A). This finding suggested that the Snhg16/miR-719/SCN1A axis may represent a therapeutic target to improve the curative effects of O_3_ in neuropathic pain.

Using a rat model of monoiodoacetate-induced osteoarthritis, Xu et al. [157] demonstrated by behavioural tests that intra-articular injection of O_3_ was able to alleviate pain. In parallel, the levels of TNF-α, IL-6, collagen-2, and autophagy-related protein LC3II were found to increase, while MMP-13 (an enzyme with a potent proteolytic capacity that is markedly expressed in osteoarthritis and rheumatoid arthritis) and p62 expression decreased, suggesting upregulation of chondrocyte autophagy. Subsequently, the same group demonstrated that in rat primary chondrocytes treated with IL-1β (i.e., in an in vitro model of osteoarthritis), O_3_ improved autophagy by activating the pathway of peroxisome proliferator-activated receptor γ (PPARγ)/mechanistic target of rapamycin (mTOR) (both involved in autophagy regulation) [158].

**Figure 3:**
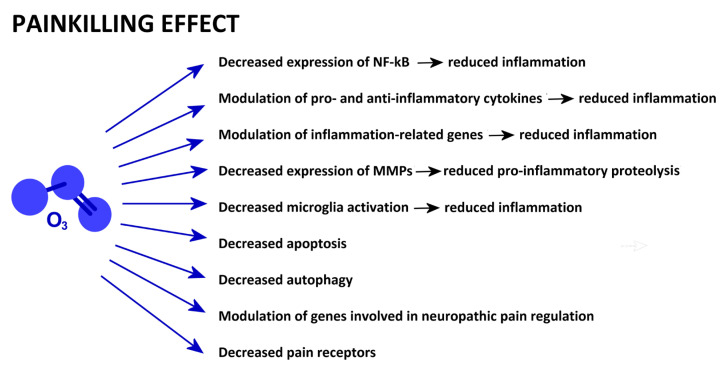
Low-dose O_3_-driven cell mechanisms involved in painkilling properties.

A schematic presentation of the mechanisms reported in this section is given in Figure 3.

## 7. Ozone and Regeneration

Many studies performed on different experimental models in vivo and in vitro have demonstrated the efficacy of O_3_ in improving tissue regeneration when applied as gas or ozonated oil/water to treat wound healing (e.g., [159,160,161,162,163]), bone healing (e.g., [164,165,166]), nerve regeneration (e.g., [167,168]), and cornea healing (e.g., [169]) in association with other therapies (drugs, hyperbaric oxygen, laser, PRP, photobiomodulation) (e.g., [170,171,172,173]).

On the other hand, studies on the biological mechanisms accounting for the regenerative potential of O_3_ are less numerous.

Some experimental evidence indicates the antioxidant and anti-inflammatory effects of O_3_ as a key contributing factor to tissue regeneration. In an in vitro model consisting of an established line of human keratinocytes, ozonated saline proved to accelerate cell wound closure, inducing the formation of 4-HNE protein adducts, increasing the protein levels of proliferating cell nuclear antigen (PCNA, which plays a key role in DNA replication and repair), activating Nrf2, and upregulating HO-1 gene expression [174].

In partially hepatectomised rats after ischaemic/reperfusion injury, O_3_ preconditioning showed a stimulatory effect on liver cell regeneration, decreased serum TNF-α levels, and maintained high levels of IL-6 [175]. The authors suggested that the reduction in TNF-α production, likely due to the antioxidant properties of O_3_, may attenuate liver injury due to ischaemia–reperfusion, while IL-6 may exert protective and pro-proliferative effects on hepatocytes.

Using a zebrafish model submitted to caudal fin resection, Hao et al. [176] demonstrated that treatment with O_3_ promoted tissue regeneration by decreasing the levels of TNF-α and modulating IL-10, IL1β, and STAT3 expression. Ozonated oil improved healing of resected caudal fin and cutaneous wounds that had been experimentally generated and inflamed in zebrafish, and O_3_ was able to reduce tissue ROS production and serum IL-6 levels [177]. O_3_ prominently accelerated healing of experimentally, surgically induced wounds in rabbits and reduced serum levels of TNF-α and IL-6 [178]. Similarly, ozonated oil markedly improved healing of skin ulcers in rats, reducing serum levels of MDA and TNF-α [179].

Beneficial effects of O_3_ administration on anastomotic healing were observed in a rat peritonitis model [180]. In animals treated with O_3_, the levels of TNF-α and IL-1β from blood serum decreased to control values in comparison to those in untreated animals, and the same trend was observed for colonic tissue levels of MDA, myeloperoxidase (MPO, an enzyme abundantly expressed in neutrophils), IL-1β, VEGF, and antigen Kiel 67 (Ki-67, a nuclear protein marker of cell proliferation). The authors therefore concluded that O_3_ promoted anastomotic healing of the colon in the presence of peritonitis through its anti-inflammatory and antioxidative properties. Accordingly, Taşdöven et al. [181] found higher hydroxyproline (a main component of collagen) and SOD levels and lower MDA levels in colonic tissue from rats undergoing O_3_ treatment before radiotherapy and colon anastomosis in comparison to untreated ones. These findings accounted for the more efficient anastomosis healing observed in O_3_-treated rats, supporting the hypothesis that this beneficial effect relies on the antioxidant properties of O_3_.

By submitting rats to sciatic nerve cut injury, Ogut et al. [73] demonstrated that post-surgical treatment with intraperitoneal injections of O_3_ improved functional and structural recovery of the nerve. Moreover, O_3_ was found to increase GPx, SOD, and CAT activity and decrease MAD levels in plasma. Since the ischaemic and inflammatory processes caused by injury induced free radicals and lipid peroxidation, the authors hypothesised that the regenerative effect of O_3_ relies on its capability to counteract the adverse effects of damage-related oxidative stress on nerve tissue. Accordingly, in a rat model of experimental spine injury, post-surgical treatment with O_3_ or O_3_ combined with methylprednisolone improved structural and functional recovery by decreasing oxidative stress (the total oxidant status was reduced), inflammation (IL-6 levels were lowered), and apoptosis [182].

Wu et al. [183] used models of osteoarthritis in vitro (a mouse chondrogenic cell line and a mouse macrophage cell line) and in vivo (mice experimentally undergoing knee osteoarthritis) and demonstrated that injectable O_3_-rich nanocomposite hydrogel promoted chondrocyte proliferation and increased the expression of collagen II and aggrecan (both components of cartilage matrix). Moreover, VEGF, IL-1β, IL-6, TNF-α, and iNOS significantly decreased, thus counteracting cartilage destruction and synovial inflammation.

The reference drug for the treatment of leishmaniasis lesions, Glucantime^®^, was associated with local and systemic ozonated saline in a mouse model [184]: O_3_ association ameliorated the drug’s leishmanicidal action and accelerated wound healing. In peripheral blood, O_3_ reduced the number of leukocytes to the level in non-infected animals; similar decreases were found for aspartate aminotransaminase and LDH (which are both released following cell damage). In peritoneal macrophages, O_3_ diminished NO secretion, increased arginase activity, and inhibited TNF-α and IL-1 secretion. It was therefore hypothesised that O_3_ downmodulates anti-inflammatory activity both locally and systemically, thus reducing cell damage and improving the healing process. Accordingly, a more recent study performed in cutaneous leishmaniasis lesions in mice under Glucantime^®^ treatment demonstrated the efficacy of ozonated oil in improving both leishmanicidal activity and wound healing, likely through an immunomodulatory action leading to decreased TNF levels and a reduced number of blood leukocytes [185].

In an in vitro study, O_3_ was demonstrated to significantly limit the degradation of explanted murine adipose tissue maintained in culture [186]. The O_3_-treated tissue showed increased levels of Nrf2 protein and overexpression of its target gene HO-1, while a metabolite analysis of culture medium demonstrated a low efflux of glycerol (a major alcohol in adipocyte lipids) and higher glutamate intake (required to maintain the function of the tricarboxylic acid cycle). These findings suggested that O_3_-driven antioxidant processes, together with the pro-adipogenic potential of O_3_ [187], may play a primary role in adipose tissue preservation, thus inviting interesting perspectives on regenerative medicine in limiting reabsorption after fat grafting.

Other experimental findings pointed to O_3_-driven stimulation of fibroblast activity.

In an in vitro study, Borges et al. [188] reported an increased migration capability of fibroblasts exposed to low O_3_ concentrations, which could contribute to the beneficial effect of O_3_ in wound healing.

By combining in vivo and in vitro investigations, Xiao et al. [189] demonstrated that ozonated oil applied to excisional wounds in mice significantly improved healing. O_3_ was found to increase the migration capability of fibroblasts, as it upregulated collagen I, α-SMA, and TGF-β1 mRNA and protein expression. O_3_ also upregulated fibronectin (a matrix glycoprotein that plays a major role in cell adhesion, growth, migration, and differentiation), vimentin (an intermediate filament protein mainly expressed in mesenchymal cells), N-cadherin (a transmembrane protein that mediates cell–cell adhesion), MMP-2, MMP-9, insulin-like growth factor (IGF) binding protein (IGFBP)-3, IGFBP-5, and IGFBP-6 (modulators of IGF signalling). Moreover, O_3_ downregulated the expression of the epithelial marker E-cadherin and the cellular senescence marker p16 while increasing the phosphorylation of some kinases involved in the epithelial–mesenchymal transition (i.e., phosphoinositide 3-kinase (PI3K), protein kinase B (Akt), mTOR) and repressing increases in TNF-α and IL-6 in injured fibroblasts. Taken together, these data demonstrated that O_3_ promotes wound healing via the PI3K/Akt/mTOR signalling pathway, which increases fibroblast migration and the epithelial–mesenchymal transition.

Interestingly, an in vitro study [141] demonstrated that O_3_ treatment is able to modulate fibroblast proliferation, surface protrusion formation (needed for cell motility), Nrf2/HO-1 expression, and IL-6 and TGF-β1 secretion depending on the activation state of cells and O_3_ concentrations, which may lead to differential fibroblast responsiveness to O_3_ in different phases of wound healing and could explain apparent inconsistencies in scientific results obtained in different experimental conditions. Moreover, O_3_ proved to be able to induce proliferation and shape modifications in non-activated fibroblasts but not in the already activated ones, suggesting that O_3_ does not pose a risk of fibroblast overactivation or dysregulation, which could potentially lead to keloid formation.

Soares et al. [190] quantified FGF-2 and α-SMA during wound healing in rats that were subcutaneously treated with O_3_, and the higher levels of FGF-2 together with the higher amount of myofibroblasts observed suggested that FGF-2 overexpression may contribute to the accelerated repairing process induced by O_3_.

Ozonated oils were found to promote acute cutaneous wound healing in the SKH1 mouse model by increasing tissue levels of 4-HNE, NF-κB, VEGF, and PCNA [191]. The authors hypothesised that the cell signalling molecule 4-HNE modulated the redox-sensitive transcription factor NF-κB, which in turn induced VEGF and PCNA expression, both of which are involved in the regulation of proliferation during wound healing. Accordingly, ozonated oil proved to accelerate healing of cutaneous wounds in guinea pigs by increasing collagen deposition and fibroblast proliferation [192]. However, increased expression of PDGF, TGF-β, and VEGF (but not FGF) was found in healing tissue. Since tissue samples were taken after 7 days from injury, the authors hypothesised that FGF may undergo upregulation in the early healing phase, supporting the notion that O_3_ provides beneficial effects in the healing process due to its stimulation of fibroblasts.

Finally, some studies suggested that the regeneration potential of O_3_ may rely on its capability of modulating and balancing the expression of catabolic and anabolic factors. In a rat model of intestinal ischaemia–reperfusion, O_3_ proved to attenuate the intestinal mucosal injury and enhance intestinal recovery by upregulating the expression of the pro-proliferation factor phosphorylated (p)-ERK while decreasing the expression of caspase-3 [193].

A rabbit model of surgery-induced osteoarthritis was used to investigate the impact of O_3_ and PRP treatment in limiting cartilage damage [194]. Associating O_3_ with PRP yielded better results, with increased expression of type II collagen and decreased levels of MMP-1 (also known as fibroblast collagenase) in cartilage tissue and significant downregulation of the mRNA expression of surgery-induced bone morphogenetic protein 2 (BMP-2, a cartilage factor that promotes matrix turnover and repair) in joint fluid. The authors concluded that PRP combined with O_3_ may prevent cartilage destruction by restoring homeostasis between anabolism and catabolism of cartilage extracellular matrix in osteoarthritis. This synergistic effect is consistent with the finding that the addition of O_3_ to PRP drastically increases the secretion of platelet-derived factors, thus enhancing the beneficial effects of PRP [114].

**Figure 4:**
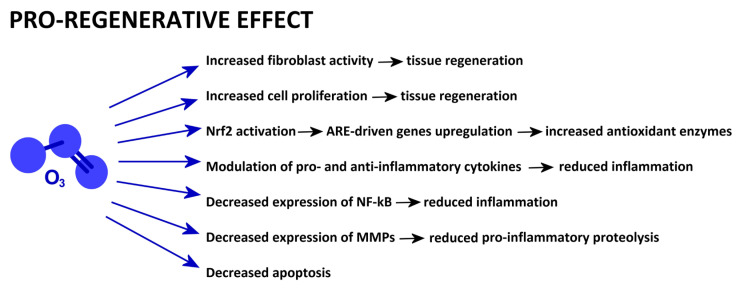
Low-dose O_3_-driven cell mechanisms involved in pro-regenerative properties.

A schematic presentation of the mechanisms reported in this section is given in Figure 4.

## 8. Ozone and Cancer

O_3_ has largely been used to limit the unpleasant side effects of radio- or chemotherapy in oncologic patients, and the mechanisms underlying its beneficial effects have been experimentally investigated both in vitro and in vivo (see the Section 4, Section 5 and Section 6). Furthermore, the potential antitumour effects of O_3_ have also been explored.

The potential anticancer effect of O_3_ was evaluated in vitro in hepatocellular carcinoma cell lines [195]. The study showed positive results since O_3_ reduced cell proliferation and migration while inducing cell cycle arrest by modulating the protein levels of p53, p21, cyclin D1, cyclin B1, cell division cycle 2 (cdc2), and CDK-4 (all involved in cell cycle regulation). O_3_ also induced ROS accumulation and GSH decreases. The authors concluded that O_3_ exerted its antitumour activity by inactivating the PI3K/AKT/NF-κB pathway. In addition to these data, a further study on hepatocellular carcinoma cells demonstrated that O_3_ treatment induced apoptosis, reduced the mitochondrial membrane potential, reduced Bcl-2 expression, and increased cleaved PARP-1, cytochrome c, caspase-3, caspase-9, and p-JNK expression, suggesting that mitochondrion-driven apoptosis may play a primary role in the O_3_ antitumour action [196]. The anticancer effectiveness of O_3_ towards liver carcinoma cells was also explored by using ozonated water [197], which induced ROS accumulation and decreased cell viability, motility, and invasion. The observed lower expression levels of p65, NF-κB, STAT3, TNF-α, IL-6, janus kinase 2 (JAK2, which is involved in cytokine signalling), Slug (a regulatory transcription factor involved in the epithelial–mesenchymal transition of cancer cells), Twist (a transcription factor implicated in cell differentiation and cancer metastasis), vimentin, MMP-2, MMP-9, and HMGB1 suggested that the inhibition of proliferation, invasion, and metastasis may occur via regulation of the HMGB1/NF-κB/STAT3 signalling pathway.

Human breast adenocarcinoma cells treated with O_3_ in vitro showed an enhanced death rate (mainly due to an increased apoptotic incidence) and a reduced migration capability together with higher mRNA expression levels of pro-apoptotic genes [198].

Treating human neuroblastoma cell lines with O_3_ resulted in an increased apoptotic rate via caspase-3 activation and PARP cleavage, with increased pro-apoptotic Bax protein. Cell cycle arrest was also increased due to altered expression and activity of the cyclin B1/CDK-1 complex and increased levels of CDK-1 and Wee1 proteins (both regulators of cell cycle progression) [199].

The efficacy of O_3_ in treating malignant liver ascites was investigated in a tumour-bearing mouse model induced by inoculation with H22 murine hepatocellular carcinoma cells [200]. O_3_ proved to reduce NET-associated guanine histone H3 and MPO in the intestinal tissue, while in the ascites, O_3_ increased the levels of circulating free DNA, IL-6, IFN-γ, MMP-9, VEGF, and TNF-α, as well as the expression of p-AMPK and SR-A proteins. O_3_ therefore reduced peritoneal fluid production by activating AMPK and upregulating SR-A phagocytosis, thus inviting interesting perspectives for its use to treat malignant ascites in hepatocellular carcinoma.

A papillomavirus-associated auricular VX2 carcinoma rabbit model was used to investigate the antitumourigenic effects of O_3_ treatment on the pneumoperitoneum [201]. Tumour tissues from treated animals exhibited increased levels of cluster of differentiation (CD)3+ T cells and enhanced expression of genes encoding receptors involved in pattern recognition (i.e., factors regulating ongoing immune responses such as CD80/CD28, CD86/CD28, CD40/CD154 (CD40L), CD4, and CD1d), together with increased levels of related downstream signalling molecules (e.g., myeloid differentiation primary response gene 88-like). IL-2, IL-10, IL-18, and IFN-γ were upregulated, while the expression of COX-2 was downregulated. It was concluded that O_3_ stimulated immune cells as a result of the induced oxidative stress.

O_3_ was found to stimulate necrotic cell death in two human pancreatic ductal adenocarcinoma cell lines [202]. Genomic analysis demonstrated that O_3_ reduced cell cycle progression by downregulating cyclins, CDKs, and the transcription factors E2F1 and E2F3 while upregulating the expression of CDKN2A. O_3_ also downregulated rat sarcoma virus (Ras)-associated pathway genes, which are correlated with various oncogenic signals, such as the proliferation, chemoresistance, and migration of pancreatic ductal adenocarcinoma cells. O_3_ treatment was also effective in reducing the expression of NF-κB, Rel, Ras homolog family member A (all involved in immunity and inflammation processes), and the PI3K/AKT pathway, thus contributing to reduced inflammation and cellular migration. Notably, the O_3_ anticancer effect was enhanced when O_3_ was administered in combination with cannabidiol, which increased the anti-tumoural effects of gemcitabine and paclitaxel.

A rat model of non-muscle invasive bladder cancer was used to study the preventive effect of O_3_ administered by intravesical instillation, which demonstrated that O_3_ increased the antioxidant response by increasing SOD levels [203].

In a mouse xenograft model of oesophageal carcinoma [204], O_3_ improved radiosensitivity by inhibiting NETs. In fact, O_3_ administration was found to lower the peripheral-blood levels of circulating free DNA (cfDNA, i.e., the degraded DNA fragments that in advanced cancer are increasingly released into body fluids), IFN-γ, MPO-DNA complexes, TNF-α, IL-6, HIF-1α, and MMP-9 while increasing p-AMPK and SR-A protein expression in tumour tissues. Accordingly, previous studies on a tongue cancer rat model [205] and on a mouse model of peritoneal carcinomatosis [206] demonstrated higher survival rates after combined treatment with radiotherapy and medical O_3_.

The anticancer potential of O_3_ on various cell lines has also been explored in association with chemotherapeutic drugs such as doxorubicin [207] or 5-fluorouracil and cisplatin [117]. In these studies, the combined treatment demonstrated enhanced anticancer efficiency by increasing apoptosis, activating the expression of TNF-α, MMP-2, and MMP-9 and decreasing IL-1β, IL-6, and IL-8 production. This invites interesting perspectives for the use of O_3_ as a complementary treatment to manage the pro-inflammatory cancer microenvironment.

O_3_ has been tested as an anticancer agent and also as ozonated olive oil encapsulated into a niosomal vesicular nanoplatform to improve transdermal penetration [208]. The permeation capability of this nanosystem was assessed ex vivo in explanted rat skin, while anticancer activity was tested in vitro in a human melanoma cell line. The authors observed higher permeation of the encapsulated form compared to free ozonated oil, with a resultant significant increase in the rate of melanoma cell death. A further nanoformulation consisting of a perfluorotributylamine core and a lipid monolayer encapsulated in a thermoresponsive hydrogel has been fabricated to load O_3_. This ozonated nanoemulsion sprayed in the surgical cavity proved to significantly reduce tumour recurrence in hepatocellular carcinoma-bearing mice by regulating the expression of several genes, such as GPx4, acyl-CoA synthetase long-chain family member 4, and CDKN1A, that promote ferroptosis and apoptosis [209]. Recently, an oleogel loaded with O_3_ and doxorubicin was fabricated for local skin tumour treatment in association with radiotherapy [210]. When mouse melanoma cells were treated in vitro with this oleogel, significant reductions in viability and GSH contents and a concomitant ROS increase were observed. In a melanoma-bearing mouse model, the oleogel decreased tumour cell proliferation, increased apoptosis and DNA damage, and induced ROS accumulation, while immune cells (e.g., CD3+ and CD8+ T cells) increased, suggesting stimulation of the immune response. Combination with radiotherapy increased the production of intracellular free radicals, thus enhancing the anticancer efficacy of the oleogel.

Doubts have been raised as to the administration of O_3_ to oncological patients since the role of Nrf2 in cancer initiation, progression, and treatment is controversial (reviewed in [211]), and interference between O_3_ and doxorubicin was observed in a human breast cancer cell line [212]. Recent studies provided experimental in vitro evidence that O_3_ at low concentrations does not induce cytokinetic effects on tumour cells or reduce drug cytotoxicity; the proliferation and motility of human cervical and breast cancer cells were not affected by O_3_ administration [213], and when O_3_ was combined with an anti-tumour agent (tamoxifen) acting through induction of oxidative stress, no reduction in drug cytotoxicity was observed as Nrf2 was not overstimulated [214].

**Figure 5:**
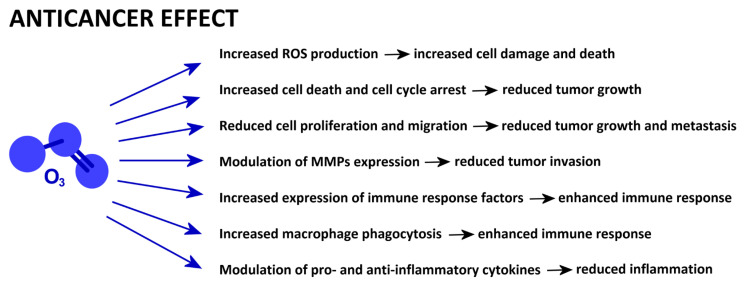
Low-dose O_3_-driven cell mechanisms involved in anticancer properties.

A schematic presentation of the mechanisms reported in this section is given in Figure 5.

## 9. Conclusions

There is still incertitude within the medical community as to the therapeutic efficacy of O_3_. This is due to several reasons, namely, the considerable heterogeneity of administration routes, procedures and protocols, the lack of internationally endorsed guidelines, the small number of pre-clinical and clinical trials, and incomplete knowledge of the O_3_ impact on biological pathways. These limitations are even more evident if medical O_3_ is compared, for instance, with long-established anti-inflammatory and painkilling pharmacological agents such as corticosteroids. In fact, the experimental and clinical literature on corticosteroid therapy is very wide (reviews in, e.g., [215,216]), providing a robust base for guidelines and protocols. Therefore, further scientific studies on medical O_3_ are needed, and experimental research is essential to mechanistically elucidate the molecular bases of the observed beneficial effects of low O_3_ concentrations on patients. This knowledge will also be crucial to improve the present clinical protocols and to design novel therapeutic strategies considering individual and pathological variability.

Some basic mechanisms accounting for the therapeutic effects of eustress-inducing low O_3_ concentrations have already been established. It has been demonstrated that O_3_ counteracts oxidative stress by activating Nrf2 and promotes cell survival by upregulating HIF-1α and inhibiting apoptosis and autophagy. O_3_ exerts its anti-inflammatory effect by modulating many cytokines, inhibiting matrix metalloproteinases, and enhancing macrophage phagocytosis. The painkilling action of O_3_ is related to these anti-inflammatory effects, to the inhibition of apoptosis and autophagy, and to the modulation of genes regulating neuropathic pain. The regenerative potential of O_3_ depends on its antioxidant, anti-inflammatory, anti-apoptotic, and pro-proliferative capabilities, as well as fibroblast stimulation. The O_3_ anticancer potential is based not only on its oxidant and anti-inflammatory properties but also on its ability to inhibit cell proliferation, invasion, and migration while promoting cell death.

Notably, some recent clinical studies supported these experimental findings by reporting that, e.g., ozonated saline/gel treatment reduced inflammation and pain in patients undergoing dental implant surgery [217]; O_3_ combined with articular injection of sodium hyaluronate decreased inflammatory factors in the joint fluid of patients affected by knee osteoarthritis [218]; intraarticular injection of O_3_ reduced pain and decreased inflammatory markers in adhesive capsulitis of the shoulder joint [219]; O_3_ treatment reduced ulcer size and healing time in diabetes-related foot ulcer [220]; and O_3_ in association with concentrated growth factors increased the secretion of growth factors and cytokines in stimulated stem cells, thus improving angiogenesis and tissue regeneration in alveolar osteitis [221]. However, in all these studies, the number of enrolled patients and analysed markers was generally small, so the clinical evidence remains limited.

As the present review showed, the current knowledge on O_3_-driven molecular effects is still incomplete, fragmentary, and seldom characterised by conflicting findings in different experimental models and conditions. This is certainly due to the high complexity of the molecular and cellular mechanisms triggered by low O_3_ concentrations and their nonuniform impact on different physiological and pathological situations. The mechanisms accounting for the effects of O_3_ in modulating mitochondrial activity and the cytoskeleton dynamic organization (which are crucial for its anticancer and regenerative properties) have rarely been investigated. Similarly, there is incomplete knowledge on the O_3_-altered molecular pathways in adipogenic differentiation (which is of great interest in regenerative medicine) and cell conduction (which is central to understanding the nerve and muscle cell interactions for pain management and restoration of sensorimotor function).

The response to the current concerns on the therapeutic efficacy of medical O_3_ administration will only come from scientific research through refined experimental investigations on the action mechanisms and from larger-scale clinical trials.

## References

[1] Barbe A., Mikhailenko S., Starikova E., Tyuterev V. (2022). High Resolution Infrared Spectroscopy in Support of Ozone Atmospheric Monitoring and Validation of the Potential Energy Function. Molecules.

[2] Forbes R.J. (1969). Martinus Van Marum. Life and Work.

[3] Rubin M.B. (2001). The history of ozone. The Schönbein period, 1839–1868. Bull. Hist. Chem..

[4] Schönbein C.F. (1840). On the Odour Accompanying Electricity and on the Probability of its Dependence on the Presence of a New Substance. Philos. Mag..

[5] Schönbein C.F. (1851). Ueber die Oxidation des Silbers und anderer Metalle durch Ozone. Ber. Verh. Nat. Ges. Basel.

[6] Schönbein C.F. (1868). Ueber des Verhalten einiger organischer Materien zum Ozon. J. Prakt. Chem..

[7] Marchand R.F. (1846). Ueber das Ozon. Ann. Phys. Chem..

[8] Schönbein C.F. (1846). Ueber die Natur des Ozons. Ann. Phys. Chem..

[9] Andrews T., Tait P.-G. (1860). On the Volumetric Relations of Ozone, and the Action of the Electrical Discharge on Oxygen and other Gases. Philos. Trans. R. Soc. London.

[10] Schönbein C.F. (1851). On Some Secondary Physiological Effects Produced by Atmospheric Electricity. Med. Chir. Trans..

[11] von Siemens W. (1857). Ueber die elektrostatische Induction und die Verzögerung des Stroms in Flaschendrähten. Poggendorff’s Ann. Phys. Chem..

[12] Fox C.B. (1873). Ozone and Antozone, Their History and Nature When, Where, Why, How Is Ozone Observed in the Atmosphere?.

[13] Kellogg J.H. (1879). Diphtheria: Its Causes, Prevention, and Proper Treatment.

[14] Viebahn-Hänsler R., León Fernández O.S. (2020). Ozone in Medicine—From Science to Guidelines and Treatment Concepts.

[15] Bocci V., Zanardi I., Valacchi G., Borrelli E., Travagli V. (2015). Validity of Oxygen-Ozone Therapy as Integrated Medication Form in Chronic Inflammatory Diseases. Cardiovasc. Hematol. Disord. Drug Targets.

[16] Oliveira Modena D.A., de Castro Ferreira R., Froes P.M., Rocha K.C. (2022). Ozone Therapy for Dermatological Conditions: A Systematic Review. J. Clin. Aesthet. Dermatol..

[17] Jeyaraman M., Jeyaraman N., Ramasubramanian S., Balaji S., Nallakumarasamy A., Patro B.P., Migliorini F. (2024). Ozone therapy in musculoskeletal medicine: A comprehensive review. Eur. J. Med. Res..

[18] Hidalgo-Tallón F.J., Torres-Morera L.M., Baeza-Noci J., Carrillo-Izquierdo M.D., Pinto-Bonilla R. (2022). Updated Review on Ozone Therapy in Pain Medicine. Front. Physiol..

[19] El Meligy O.A., Elemam N.M., Talaat I.M. (2023). Ozone Therapy in Medicine and Dentistry: A Review of the Literature. Dent. J..

[20] Veneri F., Filippini T., Consolo U., Vinceti M., Generali L. (2024). Ozone therapy in dentistry: An overview of the biological mechanisms involved (Review). Biomed. Rep..

[21] Sciorsci R.L., Lillo E., Occhiogrosso L., Rizzo A. (2020). Ozone therapy in veterinary medicine: A review. Res. Vet. Sci..

[22] Viebahn-Hänsler R., Fernández O.S.L., Fahmy Z. (2012). Ozone in medicine: The low-dose ozone concept—Guidelines and treatment strategies. Ozone Sci. Eng..

[23] Viebahn-Haensler R., Fernández O.L. (2021). Ozone in medicine. The low-dose ozone concept and its basic biochemical mechanisms of action in chronic inflammatory diseases. Int. J. Mol. Sci..

[24] Sagai M., Bocci V. (2011). Mechanisms of Action Involved in Ozone Therapy: Is healing induced via a mild oxidative stress?. Med. Gas Res..

[25] Niki E. (2016). Oxidative stress and antioxidants: Distress or eustress?. Arch. Biochem. Biophys..

[26] Sies H. (2017). Hydrogen peroxide as a central redox signaling molecule in physiological oxidative stress: Oxidative eustress. Redox Biol..

[27] Niki E. (2009). Lipid peroxidation: Physiological levels and dual biological effects. Free Radic. Biol. Med..

[28] Crawford D.R., Davies K.J. (1994). Adaptive response and oxidative stress. Environ. Health Perspect..

[29] Galiè M., Covi V., Tabaracci G., Malatesta M. (2019). The Role of Nrf2 in the Antioxidant Cellular Response to Medical Ozone Exposure. Int. J. Mol. Sci..

[30] Tricarico G., Travagli V. (2021). The Relationship between Ozone and Human Blood in the Course of a Well-Controlled, Mild, and Transitory Oxidative Eustress. Antioxidants.

[31] Bocci V., Luzzi E., Corradeschi F., Paulesu L., Rossi R., Cardaioli E., Di Simplicio P. (1993). Studies on the biological effects of ozone: 4. Cytokine production and glutathione levels in human erythrocytes. J. Biol. Regul. Homeost. Agents.

[32] Bocci V., Valacchi G., Corradeschi F., Aldinucci C., Silvestri S., Paccagnini E., Gerli R. (1998). Studies on the biological effects of ozone: 7. Generation of reactive oxygen species (ROS) after exposure of human blood to ozone. J. Biol. Regul. Homeost. Agents.

[33] Bocci V., Aldinucci C. (2006). Biochemical modifications induced in human blood by oxygenation-ozonation. J. Biochem. Mol. Toxicol..

[34] Travagli V., Zanardi I., Bernini P., Nepi S., Tenori L., Bocci V. (2010). Effects of ozone blood treatment on the metabolite profile of human blood. Int. J. Toxicol..

[35] Ciborowski M., Lipska A., Godzien J., Ferrarini A., Korsak J., Radziwon P., Tomasiak M., Barbas C. (2012). Combination of LC-MS- and GC-MS-based metabolomics to study the effect of ozonated autohemotherapy on human blood. J. Proteome Res..

[36] Inguscio C.R., Cisterna B., Carton F., Barberis E., Manfredi M., Malatesta M. (2023). Modifications of Blood Molecular Components after Treatment with Low Ozone Concentrations. Int. J. Mol. Sci..

[37] Larini A., Bianchi L., Bocci V. (2003). The ozone tolerance: I) Enhancement of antioxidant enzymes is ozone dose-dependent in Jurkat cells. Free Radic. Res..

[38] Bocci V., Aldinucci C., Mosci F., Carraro F., Valacchi G. (2007). Ozonation of human blood induces a remarkable upregulation of heme oxygenase-1 and heat stress protein-70. Mediat. Inflamm..

[39] Scassellati C., Costanzo M., Cisterna B., Nodari A., Galiè M., Cattaneo A., Covi V., Tabaracci G., Bonvicini C., Malatesta M. (2017). Effects of mild ozonisation on gene expression and nuclear domains organization in vitro. Toxicol. In Vitro.

[40] León O.S., Menéndez S., Merino N., Castillo R., Sam S., Pérez L., Cruz E., Bocci V. (1998). Ozone oxidative preconditioning: A protection against cellular damage by free radicals. Mediat. Inflamm..

[41] Candelario-Jalil E., Mohammed-Al-Dalain S., Fernández O.S., Menéndez S., Pérez-Davison G., Merino N., Sam S., Ajamieh H.H. (2001). Oxidative preconditioning affords protection against carbon tetrachloride-induced glycogen depletion and oxidative stress in rats. J. Appl. Toxicol..

[42] Guanche D., Hernandez F., Zamora Z., Alonso Y. (2010). Effect of ozone pre-conditioning on redox activity in a rat model of septic shock. Toxicol Mech. Methods.

[43] Gultekin F.A., Bakkal B.H., Guven B., Tasdoven I., Bektas S., Can M., Comert M. (2013). Effects of ozone oxidative preconditioning on radiation-induced organ damage in rats. J. Radiat Res..

[44] Aksu F., Kavaklı A., Kuloglu T., Yilmaz S., Kaya E., Akkoc R.F., Yilmaz M., Emre E., Ogetürk M. (2024). Use of Ozone Therapy and Thymoquinone in the Prevention of Formaldehyde Toxicity by Inhalation: An Experimental Study. Cureus.

[45] Borrego A., Zamora Z.B., González R., Romay C., Menéndez S., Hernández F., Montero T., Rojas E. (2004). Protection by ozone preconditioning is mediated by the antioxidant system in cisplatin-induced nephrotoxicity in rats. Mediat Inflamm..

[46] Calunga J.L., Trujillo Y., Menéndez S., Zamora Z., Alonso Y., Merino N., Montero T. (2009). Ozone oxidative post-conditioning in acute renal failure. J. Pharm. Pharmacol..

[47] Caliskan B., Guven A., Ozler M., Cayci T., Ozcan A., Bedir O., Surer I., Korkmaz A. (2011). Ozone therapy prevents renal inflammation and fibrosis in a rat model of acute pyelonephritis. Scand. J. Clin. Lab. Investig..

[48] Uğuz S., Demirer Z., Uysal B., Alp B.F., Malkoc E., Guragac A., Turker T., Ateş F., Karademir K., Ozcan A. (2016). Medical ozone therapy reduces shock wave therapy-induced renal injury. Ren. Fail..

[49] Delgado-Roche L., Hernández-Matos Y., Medina E.A., Morejón D.Á., González M.R., Martínez-Sánchez G. (2014). Ozone-Oxidative Preconditioning Prevents Doxorubicin-induced Cardiotoxicity in Sprague-Dawley Rats. Sultan Qaboos Univ. Med. J..

[50] Tasdemir S., Tasdemir C., Vardi N., Ates B., Taslidere E., Karaaslan M.G., Sapmaz H.I., Sagir M., Kurt A., Baser C.A. (2013). Effects of ozone therapy on cyclophosphamide-induced urinary bladder toxicity in rats. Clin. Investig. Med..

[51] Salem E.A., Salem N.A., Hellstrom W.J. (2017). Therapeutic effect of ozone and rutin on adriamycin-induced testicular toxicity in an experimental rat model. Andrologia.

[52] Aydogdu I., Ilbey Y.O., Coban G., Ekin R.G., Mirapoglu S.L., Cay A., Kiziltan H.S., Ekin Z.Y., Silay M.S., Semerci M.B. (2019). Does ozone administration have a protective or therapeutic effect against radiotherapy-induced testicular injury?. J. Cancer Res. Ther..

[53] Khorsandi L., Varaa N., Dadfar R., Vastegani S.M., Asadi-Fard Y., Ahangarpour A., Keshavarz-Zarjani A. (2024). The protective effect of Ozone on the mice testicular damage induced by methotrexate. JBRA Assist. Reprod..

[54] Kesik V., Uysal B., Kurt B., Kismet E., Koseoglu V. (2009). Ozone ameliorates methotrexate-induced intestinal injury in rats. Cancer Biol. Ther..

[55] Abu-Gharbieh E., Bayoumi F.A., Ahmed N.G. (2014). Alleviation of antioxidant defense system by ozonized olive oil in DNBS-induced colitis in rats. Mediat. Inflamm..

[56] Zamora Rodríguez Z.B., González Alvarez R., Guanche D., Merino N., Hernández Rosales F., Menéndez Cepero S., Alonso González Y., Schulz S. (2007). Antioxidant mechanism is involved in the gastroprotective effects of ozonized sunflower oil in ethanol-induced ulcers in rats. Mediat. Inflamm..

[57] Guven A., Gundogdu G., Sadir S., Topal T., Erdogan E., Korkmaz A., Surer I., Ozturk H. (2008). The efficacy of ozone therapy in experimental caustic esophageal burn. J. Pediatr. Surg..

[58] Al-Dalain S.M., Martínez G., Candelario-Jalil E., Menéndez S., Re L., Giuliani A., León O.S. (2001). Ozone treatment reduces markers of oxidative and endothelial damage in an experimental diabetes model in rats. Pharmacol. Res..

[59] Uysal B., Yasar M., Ersoz N., Coskun O., Kilic A., Cayc T., Kurt B., Oter S., Korkmaz A., Guven A. (2010). Efficacy of hyperbaric oxygen therapy and medical ozone therapy in experimental acute necrotizing pancreatitis. Pancreas.

[60] Kaldirim U., Uysal B., Yuksel R., Macit E., Eyi Y.E., Toygar M., Tuncer S.K., Ardic S., Arziman I., Aydin I. (2014). Ozone therapy ameliorates paraquat-induced lung injury in rats. Exp. Biol. Med..

[61] Erken H.A., Genç O., Erken G., Ayada C., Gündoğdu G., Doğan H. (2015). Ozone partially prevents diabetic neuropathy in rats. Exp. Clin. Endocrinol. Diabetes.

[62] Biçer Ş., Sayar İ., Gürsul C., Işık A., Aydın M., Peker K., Demiryilmaz İ. (2016). Use of Ozone to Treat Ileostomy Dermatitis in an Experimental Rat Model. Med. Sci. Monit..

[63] Peralta C., León O.S., Xaus C., Prats N., Jalil E.C., Planell E.S., Puig-Parellada P., Gelpí E., Roselló-Catafau J. (1999). Protective effect of ozone treatment on the injury associated with hepatic ischemia-reperfusion: Antioxidant-prooxidant balance. Free Radic. Res..

[64] Ajamieh H., Merino N., Candelario-Jalil E., Menéndez S., Martinez-Sanchez G., Re L., Giuliani A., Leon O.S. (2002). Similar protective effect of ischaemic and ozone oxidative preconditionings in liver ischaemia/reperfusion injury. Pharmacol. Res..

[65] Madej P., Plewka A., Madej J.A., Nowak M., Plewka D., Franik G., Golka D. (2007). Ozonotherapy in an induced septic shock. I. Effect of ozonotherapy on rat organs in evaluation of free radical reactions and selected enzymatic systems. Inflammation.

[66] Oztosun M., Akgul E.O., Cakir E., Cayci T., Uysal B., Ogur R., Ozcan A., Ozgurtas T., Guven A., Korkmaz A. (2012). The effects of medical ozone therapy on renal ischemia/reperfusion injury. Ren. Fail..

[67] Wang Z., Han Q., Guo Y.L., Liu X.H., Qiu T. (2018). Effect of ozone oxidative preconditioning on inflammation and oxidative stress injury in rat model of renal transplantation. Acta Cir. Bras..

[68] Guven A., Gundogdu G., Vurucu S., Uysal B., Oztas E., Ozturk H., Korkmaz A. (2009). Medical ozone therapy reduces oxidative stress and intestinal damage in an experimental model of necrotizing enterocolitis in neonatal rats. J. Pediatr. Surg..

[69] Onal O., Yetisir F., Sarer A.E., Zeybek N.D., Onal C.O., Yurekli B., Celik H.T., Sirma A., Kılıc M. (2015). Prophylactic Ozone Administration Reduces Intestinal Mucosa Injury Induced by Intestinal Ischemia-Reperfusion in the Rat. Mediat. Inflamm..

[70] Ahmed L.A., Salem H.A., Mawsouf M.N., Attia A.S., Agha A.M. (2012). Cardioprotective effects of ozone oxidative preconditioning in an in vivo model of ischemia/reperfusion injury in rats. Scand. J. Clin. Lab. Investig..

[71] Elsurer C., Onal M., Selimoglu N., Erdur O., Yilmaz M., Erdogan E., Kal O., Celik J.B., Onal O. (2020). Postconditioning Ozone Alleviates Ischemia-Reperfusion Injury and Enhances Flap Endurance in Rats. J. Investig. Surg..

[72] Koca K., Yurttas Y., Bilgic S., Cayci T., Topal T., Durusu M., Kaldirim U., Akgul E.O., Ozkan H., Yanmis I. (2010). Effect of preconditioned hyperbaric oxygen and ozone on ischemia-reperfusion induced tourniquet in skeletal bone of rats. J. Surg. Res..

[73] Ogut E., Yildirim F.B., Sarikcioglu L., Aydin M.A., Demir N. (2020). Neuroprotective Effects of Ozone Therapy After Sciatic Nerve Cut Injury. Kurume Med. J..

[74] Orlandin J.R., Pinto Santos S.I., Machado L.C., Neto P.F., Bressan F.F., Godoy Pieri N.C., Recchia K., de Paula Coutinho M., Ferreira Pinto P.A., Santucci A. (2022). Evaluation of targeted oxidative stress induced by oxygen-ozone in vitro after ischemic induction. Redox Rep..

[75] Onal M., Elsurer C., Selimoglu N., Yilmaz M., Erdogan E., Bengi Celik J., Kal O., Onal O. (2017). Ozone Prevents Cochlear Damage from Ischemia-Reperfusion Injury in Guinea Pigs. Artif. Organs..

[76] Yıldız A., Şehitoğlu M.H., Karaboğa İ., Arıkan S. (2020). Ozone treatment for high-dose systemic Steroid-Induced retinal injury. Cutan. Ocul. Toxicol..

[77] Aslan M.K., Boybeyi Ö., Şenyücel M.F., Ayva Ş., Kısa Ü., Aksoy N., Soyer T., Cesur Ö., Çakmak M. (2012). Protective effect of intraperitoneal ozone application in experimental ovarian ischemia/reperfusion injury. J. Pediatr Surg..

[78] Süzen Çaypınar S., Karakaş S., Kaya C., Sakız D., Sezer S., Ekin M. (2022). The effect of medical ozone therapy in addition to ovarian detorsion in ischemia reperfusion model. J. Obstet. Gynaecol..

[79] Ekici S., Doğan Ekici A.I., Öztürk G., Benli Aksungar F., Sinanoğlu O., Turan G., Lüleci N. (2012). Comparison of melatonin and ozone in the prevention of reperfusion injury following unilateral testicular torsion in rats. Urology.

[80] Shehata N.I., Abd-Elgawad H.M., Mawsouf M.N., Shaheen A.A. (2012). The potential role of ozone in ameliorating the age-related biochemical changes in male rat cerebral cortex. Biogerontology.

[81] Safwat M.H., El-Sawalhi M.M., Mausouf M.N., Shaheen A.A. (2014). Ozone ameliorates age-related oxidative stress changes in rat liver and kidney: Effects of pre- and post-ageing administration. Biochemistry.

[82] El-Sawalhi M.M., Darwish H.A., Mausouf M.N., Shaheen A.A. (2013). Modulation of age-related changes in oxidative stress markers and energy status in the rat heart and hippocampus: A significant role for ozone therapy. Cell Biochem. Funct..

[83] Elkholy W.B., Al-Gholam M.A. (2018). Role of medical ozone in attenuating age-related changes in the rat cerebellum. Microscopy.

[84] Pecorelli A., Bocci V., Acquaviva A., Belmonte G., Gardi C., Virgili F., Ciccoli L., Valacchi G. (2013). NRF2 activation is involved in ozonated human serum upregulation of HO-1 in endothelial cells. Toxicol. Appl. Pharmacol..

[85] Re L., Martínez-Sánchez G., Bordicchia M., Malcangi G., Pocognoli A., Morales-Segura M.A., Rothchild J., Rojas A. (2014). Is ozone pre-conditioning effect linked to Nrf2/EpRE activation pathway in vivo? A preliminary result. Eur. J. Pharmacol..

[86] Galiè M., Costanzo M., Nodari A., Boschi F., Calderan L., Mannucci S., Covi V., Tabaracci G., Malatesta M. (2018). Mild ozonisation activates antioxidant cell response by the Keap1/Nrf2 dependent pathway. Free Radic. Biol. Med..

[87] Siniscalco D., Trotta M.C., Brigida A.L., Maisto R., Luongo M., Ferraraccio F., D′Amico M., Di Filippo C. (2018). Intraperitoneal Administration of Oxygen/Ozone to Rats Reduces the Pancreatic Damage Induced by Streptozotocin. Biology.

[88] Ding S., Duanmu X., Xu L., Zhu L., Wu Z. (2023). Ozone pretreatment alleviates ischemiareperfusion injury-induced myocardial ferroptosis by activating the Nrf2/Slc7a11/Gpx4 axis. Biomed. Pharmacother..

[89] Qiu T., Wang Z.S., Liu X.H., Chen H., Zhou J.Q., Chen Z.Y., Wang M., Jiang G.J., Wang L., Yu G. (2017). Effect of ozone oxidative preconditioning on oxidative stress injury in a rat model of kidney transplantation. Exp. Ther. Med..

[90] Yu G., Liu X., Chen Z., Chen H., Wang L., Wang Z., Qiu T., Weng X. (2016). Ozone therapy could attenuate tubulointerstitial injury in adenine-induced CKD rats by mediating Nrf2 and NF-κB. Iran J. Basic Med. Sci..

[91] Wang Z., Zhang A., Meng W., Wang T., Li D., Liu Z., Liu H. (2018). Ozone protects the rat lung from ischemia-reperfusion injury by attenuating NLRP3-mediated inflammation, enhancing Nrf2 antioxidant activity and inhibiting apoptosis. Eur. J. Pharmacol..

[92] Zhu F., Ding S., Liu Y., Wang X., Wu Z. (2024). Ozone-mediated cerebral protection: Unraveling the mechanism through ferroptosis and the NRF2/SLC7A11/GPX4 signaling pathway. J. Chem. Neuroanat..

[93] Mallok A., Vaillant J.D., Soto M.T., Viebahn-Hänsler R., Viart Mde L., Pérez A.F., Cedeño R.I., Fernández O.S. (2015). Ozone protective effects against PTZ-induced generalized seizures are mediated by reestablishment of cellular redox balance and A1 adenosine receptors. Neurol. Res..

[94] León Fernández O.S., Ajamieh H.H., Berlanga J., Menéndez S., Viebahn-Hánsler R., Re L., Carmona A.M. (2008). Ozone oxidative preconditioning is mediated by A1 adenosine receptors in a rat model of liver ischemia/ reperfusion. Transpl. Int..

[95] Chen H., Xing B., Liu X., Zhan B., Zhou J., Zhu H., Chen Z. (2008). Ozone oxidative preconditioning protects the rat kidney from reperfusion injury: The role of nitric oxide. J. Surg. Res..

[96] Chen H., Xing B., Liu X., Zhan B., Zhou J., Zhu H., Chen Z. (2008). Similarities between ozone oxidative preconditioning and ischemic preconditioning in renal ischemia/reperfusion injury. Arch. Med. Res..

[97] Ozkan H., Ekinci S., Uysal B., Akyildiz F., Turkkan S., Ersen O., Koca K., Seven M.M. (2015). Evaluation and comparison of the effect of hypothermia and ozone on ischemia-reperfusion injury of skeletal muscle in rats. J. Surg. Res..

[98] Wang L., Chen Z., Liu Y., Du Y., Liu X. (2018). Ozone oxidative postconditioning inhibits oxidative stress and apoptosis in renal ischemia and reperfusion injury through inhibition of MAPK signaling pathway. Drug Des. Devel. Ther..

[99] Jiang B., Su Y., Chen Q., Dong L., Zhou W., Li H., Wang Y. (2020). Protective Effects of Ozone Oxidative Postconditioning on Long-term Injury After Renal Ischemia/Reperfusion in Rat. Transplant. Proc..

[100] Zhu L., Ding S., Xu L., Wu Z. (2022). Ozone treatment alleviates brain injury in cerebral ischemic rats by inhibiting the NF-κB signaling pathway and autophagy. Cell Cycle.

[101] Xu L., Zhu L., Liu P., Wu Z., Zou Z. (2021). Ozone induces tolerance against cardiomyocytes oxygen-glucose deprivation/reperfusion through inhibition of autophagy pathway. Exp. Ther. Med..

[102] Wang R., Liu F., Huang P., Zhang Y., He J., Pang X., Zhang D., Guan Y. (2022). Ozone preconditioning protects rabbit heart against global ischemia-reperfusion injury in vitro by up-regulating HIF-1α. Biomed. Pharmacother..

[103] Madej P., Plewka A., Madej J.A., Plewka D., Mroczka W., Wilk K., Dobrosz Z. (2007). Ozone therapy in induced endotoxemic shock. II. The effect of ozone therapy upon selected histochemical reactions in organs of rats in endotoxemic shock. Inflammation.

[104] Nasezadeh P., Shahi F., Fridoni M., Seydi E., Izadi M., Salimi A. (2017). Moderate O_3_/O_2_ therapy enhances enzymatic and non-enzymatic antioxidant in brain and cochlear that protects noise-induced hearing loss. Free Radic. Res..

[105] Inguscio C.R., Dalla Pozza E., Dando I., Boschi F., Tabaracci G., Angelini O., Picotti P.M., Malatesta M., Cisterna B. (2023). Mitochondrial Features of Mouse Myoblasts Are Finely Tuned by Low Doses of Ozone: The Evidence In Vitro. Int. J. Mol. Sci..

[106] Oliveira M.M., Correia S., Peirone C., Magalhães M., Oliveira P., Peixoto F. (2024). Impact of ozone therapy on mouse liver mitochondrial function and antioxidant system. Biochimie.

[107] Bocci V., Luzzi E., Corradeschi F., Paulesu L., Di Stefano A. (1993). Studies on the biological effects of ozone: 3. An attempt to define conditions for optimal induction of cytokines. Lymphokine Cytokine Res..

[108] Bocci V., Luzzi E., Corradeschi F., Silvestri S. (1994). Studies on the biological effects of ozone: 6. Production of transforming growth factor 1 by human blood after ozone treatment. J. Biol. Regul. Homeost. Agents.

[109] Bocci V., Valacchi G., Corradeschi F., Fanetti G. (1998). Studies on the biological effects of ozone: 8. Effects on the total antioxidant status and on interleukin-8 production. Mediat. Inflamm..

[110] Bocci V., Paulesu L. (1990). Studies on the biological effects of ozone 1. Induction of interferon gamma on human leucocytes. Haematologica.

[111] Paulesu L., Luzzi E., Bocci V. (1991). Studies on the biological effects of ozone: 2. Induction of tumor necrosis factor (TNF-alpha) on human leucocytes. Lymphokine Cytokine Res..

[112] Larini A., Bocci V. (2005). Effects of ozone on isolated peripheral blood mononuclear cells. Toxicol. In Vitro.

[113] Valacchi G., Bocci V. (1999). Studies on the biological effects of ozone: 10. Release of factors from ozonated human platelets. Mediat. Inflamm..

[114] Inguscio C.R., Cisterna B., Lacavalla M.A., Donati F., Angelini O., Tabaracci G., Malatesta M. (2023). Ozone and procaine increase secretion of platelet-derived factors in platelet-rich plasma. Eur. J. Histochem..

[115] Bocci V., Luzzi E., Corradeschi F., Paulesu L. (1993). Studies on the biological effects of ozone: 5. Evaluation of immunological parameters and tolerability in normal volunteers receiving ambulatory autohaemotherapy. Biotherapy.

[116] Valacchi G., Bocci V. (2000). Studies on the biological effects of ozone: 11. Release of factors from human endothelial cells. Mediat. Inflamm..

[117] Simonetti V., Quagliariello V., Giustetto P., Franzini M., Iaffaioli R.V. (2017). Association of Ozone with 5-Fluorouracil and Cisplatin in Regulation of Human Colon Cancer Cell Viability: In Vitro Anti-Inflammatory Properties of Ozone in Colon Cancer Cells Exposed to Lipopolysaccharides. Evid. Based Complement. Alternat. Med..

[118] Cho K.H., Kim J.E., Bahuguna A., Kang D.J. (2023). Long-Term Supplementation of Ozonated Sunflower Oil Improves Dyslipidemia and Hepatic Inflammation in Hyperlipidemic Zebrafish: Suppression of Oxidative Stress and Inflammation against Carboxymethyllysine Toxicity. Antioxidants.

[119] Kato Y., Sakoh M., Nagai T., Yoshida A., Ishida H., Inoue N., Yanagita T., Nagao K. (2024). Ozonated Olive Oil Intake Attenuates Hepatic Steatosis in Obese db/db Mice. J. Oleo Sci..

[120] Chen H., Xing B., Liu X., Zhan B., Zhou J., Zhu H., Chen Z. (2008). Ozone oxidative preconditioning inhibits inflammation and apoptosis in a rat model of renal ischemia/reperfusion injury. Eur. J. Pharmacol..

[121] Güçlü A., Erken H.A., Erken G., Dodurga Y., Yay A., Özçoban Ö., Şimşek H., Akçılar A., Koçak F.E. (2016). The effects of ozone therapy on caspase pathways, TNF-α, and HIF-1α in diabetic nephropathy. Int. Urol. Nephrol..

[122] Sancak E.B., Turkön H., Çukur S., Erimsah S., Akbas A., Gulpinar M.T., Toman H., Sahin H., Uzun M. (2016). Major Ozonated Autohemotherapy Preconditioning Ameliorates Kidney Ischemia-Reperfusion Injury. Inflammation.

[123] Vaillant J.D., Fraga A., Díaz M.T., Mallok A., Viebahn-Hänsler R., Fahmy Z., Barberá A., Delgado L., Menéndez S., Fernández O.S. (2013). Ozone oxidative postconditioning ameliorates joint damage and decreases pro-inflammatory cytokine levels and oxidative stress in PG/PS-induced arthritis in rats. Eur. J. Pharmacol..

[124] Tartari A.P.S., Moreira F.F., Pereira M.C.D.S., Carraro E., Cidral-Filho F.J., Salgado A.I., Kerppers I.I. (2020). Anti-inflammatory Effect of Ozone Therapy in an Experimental Model of Rheumatoid Arthritis. Inflammation.

[125] Yamanel L., Kaldirim U., Oztas Y., Coskun O., Poyrazoglu Y., Durusu M., Cayci T., Ozturk A., Demirbas S., Yasar M. (2011). Ozone therapy and hyperbaric oxygen treatment in lung injury in septic rats. Int. J. Med. Sci..

[126] Aslaner A., Çakır T., Tekeli S.Ö., Avcı S., Doğan U., Tekeli F., Soylu H., Akyüz C., Koç S., Üstünel İ. (2016). Medical ozone treatment ameliorates the acute distal colitis in rat. Acta Cir. Bras..

[127] Uysal B., Demirbag S., Poyrazoglu Y., Cayci T., Yesildaglar N., Guven A., Sürer I., Korkmaz A. (2012). Medical ozone therapy decreases postoperative uterine adhesion formation in rats. Arch. Gynecol. Obstet..

[128] Wei A., Feng H., Jia X.M., Tang H., Liao Y.Y., Li B.R. (2018). Ozone therapy ameliorates inflammation and endometrial injury in rats with pelvic inflammatory disease. Biomed. Pharmacother..

[129] Suzuki N., Hirano M., Shinozuka Y., Kawai K., Okamoto Y., Isobe N. (2022). Effects of ozonized glycerin on inflammation of mammary glands induced by intramammary lipopolysaccharide infusion in goats. Anim. Sci. J..

[130] Xie T.Y., Yan W., Lou J., Chen X.Y. (2016). Effect of ozone on vascular endothelial growth factor (VEGF) and related inflammatory cytokines in rats with diabetic retinopathy. Genet. Mol. Res..

[131] Kaya A., Sonmez M., Kar T., Haholu A., Yildirim Y., Müftüoğlu T., Ünal M.H. (2017). Efficiency of Ozone Therapy in a Rat Model of Experimental Uveitis. Ocul. Immunol. Inflamm..

[132] Lu J., Chen M., Gao L., Cheng Q., Xiang Y., Huang J., Wu K., Huang J., Li M. (2018). A preliminary study on topical ozonated oil in the therapeutic management of atopic dermatitis in murine. J. Dermatolog. Treat..

[133] Salem N.A., Assaf N., Ismail M.F., Khadrawy Y.A., Samy M. (2016). Ozone Therapy in Ethidium Bromide-Induced Demyelination in Rats: Possible Protective Effect. Cell. Mol. Neurobiol..

[134] Chen Z., Liu X., Yu G., Chen H., Wang L., Wang Z., Qiu T., Weng X. (2016). Ozone therapy ameliorates tubulointerstitial inflammation by regulating TLR4 in adenine-induced CKD rats. Ren. Fail..

[135] Xu L., Wang C., Zou Z., Wu Z. (2021). Ozone Attenuated H9c2 Cell Injury Induced by Doxorubicin. J. Cardiovasc. Pharmacol..

[136] Simonetti V., Franzini M., Iaffaioli R.V., Pandolfi S.V.L., Quagliariello V. (2018). Anti-inflammatory effects of ozone in human melanoma cells and its modulation of tumour microenvironment. Int. J. Adv. Res..

[137] Simonetti V., Quagliariello V., Franzini M., Iaffaioli R.V., Maurea N., Valdenassi L. (2019). Ozone Exerts Cytoprotective and Anti-Inflammatory Effects in Cardiomyocytes and Skin Fibroblasts after Incubation with Doxorubicin. Evid Based Complement Alternat. Med..

[138] Sun W., Pei L. (2012). Ozone preconditioning and exposure to ketamine attenuates hepatic inflammation in septic rats. Arch. Med. Sci..

[139] Kucukgul A., Erdogan S., Gonenci R., Ozan G. (2016). Beneficial effects of nontoxic ozone on H_2_O_2_-induced stress and inflammation. Biochem. Cell Biol..

[140] Bertuccio M.P., Rizzo V., Arena S., Trainito A., Montalto A.S., Caccamo D., Currò M., Romeo C., Impellizzeri P. (2023). Ozoile Reduces the LPS-Induced Inflammatory Response in Colonic Epithelial Cells and THP-1 Monocytes. Curr. Issues Mol. Biol..

[141] Cisterna B., Costanzo M., Lacavalla M.A., Galiè M., Angelini O., Tabaracci G., Malatesta M. (2021). Low Ozone Concentrations Differentially Affect the Structural and Functional Features of Non-Activated and Activated Fibroblasts In Vitro. Int. J. Mol. Sci..

[142] Lacavalla M.A., Inguscio C.R., Cisterna B., Bernardi P., Costanzo M., Galiè M., Scambi I., Angelini O., Tabaracci G., Malatesta M. (2022). Ozone at low concentration modulates microglial activity in vitro: A multimodal microscopy and biomolecular study. Microsc. Res. Tech..

[143] Yu G., Bai Z., Chen Z., Chen H., Wang G., Wang G., Liu Z. (2017). The NLRP3 inflammasome is a potential target of ozone therapy aiming to ease chronic renal inflammation in chronic kidney disease. Int. Immunopharmacol..

[144] Wang D., Liu Y., Zong X., Yan S., Lu J. (2023). Ozonated triglyceride protects against septic lethality via preventing the activation of NLRP3 inflammasome. Zhong Nan Da Xue Xue Bao Yi Xue Ban..

[145] Yan C., Zhang Y., Jin L., Liu X., Zhu X., Li Q., Wang Y., Hu L., He X., Bao H. (2024). Medical ozone alleviates acute lung injury by enhancing phagocytosis targeting NETs via AMPK/SR-A1 axis. J. Biomed. Res..

[146] Kim S.Y., Lee J.O., Lee S., Heo J., Cho K.H., Bahuguna A., Yoo K.H., Kim B.J. (2024). Ozonated Sunflower Oil (OSO) Alleviates Inflammatory Responses in Oxazolone-Induced Atopic Dermatitis (AD)-Like Mice and LPS-Treated RAW 264.7 Cells. J. Microbiol. Biotechnol..

[147] Fuccio C., Luongo C., Capodanno P., Giordano C., Scafuro M.A., Siniscalco D., Lettieri B., Rossi F., Maione S., Berrino L. (2009). A single subcutaneous injection of ozone prevents allodynia and decreases the over-expression of pro-inflammatory caspases in the orbito-frontal cortex of neuropathic mice. Eur. J. Pharmacol..

[148] Murphy K., Elias G., Steppan J., Boxley C., Balagurunathan K., Victor X., Meaders T., Muto M. (2016). Percutaneous Treatment of Herniated Lumbar Discs with Ozone: Investigation of the Mechanisms of Action. J. Vasc. Interv. Radiol..

[149] Wang J., Wu M., Lin X., Li Y., Fu Z. (2018). Low-Concentration Oxygen/Ozone Treatment Attenuated Radiculitis and Mechanical Allodynia via PDE2A-cAMP/cGMP-NF-κB/p65 Signaling in Chronic Radiculitis Rats. Pain Res. Manag..

[150] Wu M.Y., Xing C.Y., Wang J.N., Li Y., Lin X.W., Fu Z.J. (2018). Therapeutic dosage of ozone inhibits autophagy and apoptosis of nerve roots in a chemically induced radiculoneuritis rat model. Eur. Rev. Med. Pharmacol. Sci..

[151] Lu L., Pan C., Chen L., Hu L., Wang C., Han Y., Yang Y., Cheng Z., Liu W.T. (2017). AMPK activation by peri-sciatic nerve administration of ozone attenuates CCI-induced neuropathic pain in rats. J. Mol. Cell Biol..

[152] Fan W., Liu C., Chen D., Xu C., Qi X., Zhang A., Zhu X., Liu Y., Wang L., Hao L. (2023). Ozone alleviates MSU-induced acute gout pain via upregulating AMPK/GAS6/MerTK/SOCS3 signaling pathway. J. Transl. Med..

[153] Zhang X.T., Zong L.J., Jia R.M., Qin X.M., Ruan S.R., Lu L.L., Wang P., Hu L., Liu W.T., Yang Y. (2023). Ozone attenuates chemotherapy-induced peripheral neuropathy via upregulating the AMPK-SOCS3 axis. J. Cancer Res. Ther..

[154] Zhang W., Wang F., Zhang L., Sun T., Fu Z. (2021). Intrathecal injection of ozone alleviates CCI-induced neuropathic pain via the GluR6-NF-κB/p65 signalling pathway in rats. Mol. Med. Rep..

[155] Yang X., Chen C., Wang K., Chen M., Wang Y., Chen Z., Zhao W., Ou S. (2023). Elucidating the molecular mechanisms of ozone therapy for neuropathic pain management by integrated transcriptomic and metabolomic approach. Front. Genet..

[156] Yue J., Wang Q., Zhao W., Wu B., Ni J. (2024). Long non-coding RNA Snhg16 Lessens Ozone Curative Effect on Chronic Constriction Injury mice via microRNA-719/SCN1A axis. Mol. Biotechnol..

[157] Xu W., Zhao X., Sun P., Zhang C., Fu Z., Zhou D. (2020). The effect of medical ozone treatment on cartilage chondrocyte autophagy in a rat model of osteoarthritis. Am. J. Transl. Res..

[158] Sun P., Xu W., Zhao X., Zhang C., Lin X., Gong M., Fu Z. (2022). Ozone induces autophagy by activating PPARγ/mTOR in rat chondrocytes treated with IL-1β. J. Orthop. Surg. Res..

[159] Lelyanov A.D., Sergienko V.I., Ivliev N.V., Emel’yanov V.V., Guseva E.D. (2004). Effects of sodium hypochlorite and ozone on healing of intestinal anastomosis in simulated strangulation colorectal obstruction. Bull. Exp. Biol. Med..

[160] Valacchi G., Lim Y., Belmonte G., Miracco C., Zanardi I., Bocci V., Travagli V. (2011). Ozonated sesame oil enhances cutaneous wound healing in SKH1 mice. Wound Repair Regen..

[161] Eroglu Z.T., Kurtis B., Altug H.A., Sahin S., Tuter G., Baris E. (2018). Effect of topical ozonetherapy on gingival wound healing in pigs: Histological and immuno-histochemical analysis. J. Appl. Oral. Sci..

[162] Pchepiorka R., Moreira M.S., Lascane N.A.D.S., Catalani L.H., Allegrini S., de Lima N.B., Gonçalves E.F. (2020). Effect of ozone therapy on wound healing in the buccal mucosa of rats. Arch Oral Biol..

[163] Karakaya E., Akdur A., Ayvazoğlu Soy E., Araz C., Ok Atilgan A., Özturan Özer E., Şençelikel T., Haberal M. (2021). Effect of Subcutaneous Topical Ozone Therapy on Second-Degree Burn Wounds in Rats: An Experimental Study. J. Burn Care Res..

[164] Ozdemir H., Toker H., Balcı H., Ozer H. (2013). Effect of ozone therapy on autogenous bone graft healing in calvarial defects: A histologic and histometric study in rats. J. Periodontal Res..

[165] Alpan A.L., Toker H., Ozer H. (2016). Ozone Therapy Enhances Osseous Healing in Rats with Diabetes With Calvarial Defects: A Morphometric and Immunohistochemical Study. J. Periodontol..

[166] Arıcı G., Şençimen M., Coşkun A.T., Altuğ H.A., Güreşci S., Çelik H.H., Uzuner M.B., Ocak M. (2022). Evaluation of the Efficiency of the Graft Material Combined with Ozonized Blood in Maxillary Sinus Lifting Applications in Rabbits. J. Maxillofac. Oral Surg..

[167] Ozbay I., Ital I., Kucur C., Akcılar R., Deger A., Aktas S., Oghan F. (2017). Effects of ozone therapy on facial nerve regeneration. Braz. J. Otorhinolaryngol..

[168] Kızılay Z., Kahraman Çetin N., Aksel M., Abas B.İ., Aktaş S., Erken H.A., Topçu A., Yılmaz A., Yenisey C. (2019). Ozone Partially Decreases Axonal and Myelin Damage in an Experimental Sciatic Nerve Injury Model. J. Investig. Surg..

[169] Aydoğan S., Erol H., Baran M. (2023). Effects of ozone therapy on acidic corneal burns in rats. Vet. Res. Forum.

[170] Kan B., Sencimen M., Bayar G.R., Korkusuz P., Coskun A.T., Korkmaz A., Bahador E., Zerener T. (2015). Histomorphometric and microtomographic evaluation of the effects of hyperbaric oxygen and systemic ozone, used alone and in combination, on calvarial defect healing in rats. J. Oral Maxillofac. Surg..

[171] Ozturk O., Tezcan A.H., Adali Y., Yıldırım C.H., Aksoy O., Yagmurdur H., Bilge A. (2016). Effect of ozone and methylprednisolone treatment following crush type sciatic nerve injury. Acta Cir. Bras..

[172] Yuca Y., Yucesoy T., Tok O.E., Alkan A. (2020). The efficiency of ozone therapy and low-level laser therapy in rat facial nerve injury. J. Craniomaxillofac. Surg..

[173] Gurger M., Once G., Yilmaz E., Demir S., Calik I., Say Y., Kavakli A., Key S., Gurbuz M.U., Bingollu O. (2021). The effect of the platelet-rich plasma and ozone therapy on tendon-to-bone healing in the rabbit rotator cuff repair model. J. Orthop. Surg. Res..

[174] Valacchi G., Sticozzi C., Zanardi I., Belmonte G., Cervellati F., Bocci V., Travagli V. (2016). Ozone mediators effect on „in vitro” scratch wound closure. Free Radic. Res..

[175] Gultekin F.A., Cakmak G.K., Turkcu U.O., Yurdakan G., Demir F.E., Comert M. (2013). Effects of ozone oxidative preconditioning on liver regeneration after partial hepatectomy in rats. J. Investig. Surg..

[176] Hao K., Li Y., Feng J., Zhang W., Zhang Y., Ma N., Zeng Q., Pang H., Wang C., Xiao L. (2015). Ozone promotes regeneration by regulating the inflammatory response in zebrafish. Int. Immunopharmacol..

[177] Cho K.H., Kim J.E., Bahuguna A., Kang D.J. (2023). Ozonated Sunflower Oil Exerted Potent Anti-Inflammatory Activities with Enhanced Wound Healing and Tissue Regeneration Abilities against Acute Toxicity of Carboxymethyllysine in Zebrafish with Improved Blood Lipid Profile. Antioxidants.

[178] Sahin H., Simsek T., Turkon H., Kalkan Y., Ozkul F., Ozkan M.T., Erbas M., Altinisik U., Demiraran Y. (2016). The acute effects of preoperative ozone theraphy on surgical wound healing. Acta Cir. Bras..

[179] Kesik V., Yuksel R., Yigit N., Saldir M., Karabacak E., Erdem G., Babacan O., Gulgun M., Korkmazer N., Bayrak Z. (2016). Ozone Ameliorates Doxorubicine-Induced Skin Necrosis—*Results* from an animal model. Int. J. Low Extrem. Wounds.

[180] Çakır T., Aslaner A., Tekeli S.Ö., Avcı S., Doğan U., Tekeli F., Soylu H., Akyüz C., Koç S., Üstünel İ. (2016). Effect of ozone on colon anastomoses in rat peritonitis model. Acta Cir. Bras..

[181] Taşdöven İ., Emre A.U., Gültekin F.A., Öner M.Ö., Bakkal B.H., Türkcü Ü.Ö., Gün B.D., Taşdöven G. (2019). Effects of ozone preconditioning on recovery of rat colon anastomosis after preoperative radiotherapy. Adv. Clin. Exp. Med..

[182] Gürkan G., Sayin M., Kizmazoglu C., Erdogan M.A., Yigitturk G., Erbak Yilmaz H., Uzunoglu I., Kaya I., Yuceer N. (2020). Evaluation of the neuroprotective effects of ozone in an experimental spine injury model. J. Neurosurg. Spine.

[183] Wu H., Wang J., Lin Y., He W., Hou J., Deng M., Chen Y., Liu Q., Lu A., Cui Z. (2024). Injectable Ozone-Rich Nanocomposite Hydrogel Loaded with D-Mannose for Anti-Inflammatory and Cartilage Protection in Osteoarthritis Treatment. Small.

[184] Cabral I.L., Utzig S.L., Banhuk F.W., Staffen I.V., Loth E.A., de Amorim J.P.A., Negretti F., Gandra R.F., Ayala T.S., Menolli R.A. (2020). Aqueous ozone therapy improves the standard treatment of leishmaniasis lesions in animals leading to local and systemic alterations. Parasitol. Res..

[185] Pivotto A.P., de Souza Lima L.B., Michelon A., Ferreira C.Z.P., Gandra R.F., Ayala T.S., Menolli R.A. (2024). Topical application of ozonated sunflower oil accelerates the healing of lesions of cutaneous leishmaniasis in mice under meglumine antimoniate treatment. Med. Microbiol. Immunol..

[186] Cisterna B., Costanzo M., Nodari A., Galiè M., Zanzoni S., Bernardi P., Covi V., Tabaracci G., Malatesta M. (2020). Ozone Activates the Nrf2 Pathway and Improves Preservation of Explanted Adipose Tissue In Vitro. Antioxidants.

[187] Costanzo M., Boschi F., Carton F., Conti G., Covi V., Tabaracci G., Sbarbati A., Malatesta M. (2018). Low ozone concentrations promote adipogenesis in human adipose-derived adult stem cells. Eur. J. Histochem..

[188] Borges G.Á., Elias S.T., da Silva S.M., Magalhães P.O., Macedo S.B., Ribeiro A.P., Guerra E.N. (2017). In vitro evaluation of wound healing and antimicrobial potential of ozone therapy. J. Craniomaxillofac. Surg..

[189] Xiao W., Tang H., Wu M., Liao Y., Li K., Li L., Xu X. (2017). Ozone oil promotes wound healing by increasing the migration of fibroblasts via PI_3_K/Akt/mTOR signaling pathway. Biosci. Rep..

[190] Soares C.D., Morais T.M.L., Araújo R.M.F.G., Meyer P.F., Oliveira E.A.F., Silva R.M.V., Carreiro E.M., Carreiro E.P., Belloco V.G., Mariz B.A.L.A. (2019). Effects of subcutaneous injection of ozone during wound healing in rats. Growth Factors.

[191] Valacchi G., Zanardi I., Lim Y., Belmonte G., Miracco C., Sticozzi C., Bocci V., Travagli V. (2013). Ozonated oils as functional dermatological matrices: Effects on the wound healing process using SKH1 mice. Int. J. Pharm..

[192] Kim H.S., Noh S.U., Han Y.W., Kim K.M., Kang H., Kim H.O., Park Y.M. (2009). Therapeutic effects of topical application of ozone on acute cutaneous wound healing. J. Korean Med. Sci..

[193] Haj B., Sukhotnik I., Shaoul R., Pollak Y., Coran A.G., Bitterman A., Matter I. (2014). Effect of ozone on intestinal recovery following intestinal ischemia-reperfusion injury in a rat. Pediatr. Surg. Int..

[194] Huang P., Wang R., Pang X., Yang Y., Guan Y., Zhang D. (2022). Platelet-rich plasma combined with ozone prevents cartilage destruction and improves weight-bearing asymmetry in a surgery-induced osteoarthritis rabbit model. Ann. Palliat. Med..

[195] Li J., Zeng T., Tang S., Zhong M., Huang Q., Li X., He X. (2021). Medical ozone induces proliferation and migration inhibition through ROS accumulation and PI3K/AKT/NF-κB suppression in human liver cancer cells in vitro. Clin. Transl. Oncol..

[196] Tang S., Xu B., Li J., Zhong M., Hong Z., Zhao W., Zeng T., He X. (2021). Ozone induces BEL7402 cell apoptosis by increasing reactive oxygen species production and activating JNK. Ann. Transl. Med..

[197] Tang S., Xu B., Pang H., Xiao L., Mei Q., He X. (2023). Ozonated Water Inhibits Hepatocellular Carcinoma Invasion and Metastasis by Regulating the HMGB1/NF-κB/STAT3 Signaling Pathway. J. Hepatocell. Carcinoma.

[198] Yıldırım M., Erkişi S., Yılmaz H., Ünsal N., İnaç E., Tanrıver Y., Koçak P. (2022). The apoptotic effect of ozone therapy on mitochondrial activity of highly metastatic breast cancer cell line MDA-MB-231 using in vitro approaches. J. Interv. Med..

[199] Cannizzaro A., Verga Falzacappa C.V., Martinelli M., Misiti S., Brunetti E., Bucci B. (2007). O_2/3_ exposure inhibits cell progression affecting cyclin B1/cdk1 activity in SK-N-SH while induces apoptosis in SK-N-DZ neuroblastoma cells. J. Cell Physiol..

[200] Han F., Guo J., Mu M., Bian K., Cui Z., Duan Q., Ma J., Jin L., Liu W., Chen F. (2023). Mechanism of ozone alleviation of malignant ascites in hepatocellular carcinoma through the inhibition of neutrophil extracellular traps. PNAS Nexus.

[201] Rossmann A., Mandic R., Heinis J., Höffken H., Küssner O., Kinscherf R., Weihe E., Bette M. (2014). Intraperitoneal oxidative stress in rabbits with papillomavirus-associated head and neck cancer induces tumoricidal immune response that is adoptively transferable. Clin. Cancer Res..

[202] Luongo M., Marinelli O., Zeppa L., Aguzzi C., Morelli M.B., Amantini C., Frassineti A., di Costanzo M., Fanelli A., Santoni G. (2020). Cannabidiol and Oxygen-Ozone Combination Induce Cytotoxicity in Human Pancreatic Ductal Adenocarcinoma Cell Lines. Cancers.

[203] Teke K., Ozkan T.A., Cebeci O.O., Yilmaz H., Keles M.E., Ozkan L., Dillioglugil M.O., Yildiz D.K., Dillioglugil O. (2017). Preventive effect of intravesical ozone supplementation on n-methyl-n-nitrosourea-induced non-muscle invasive bladder cancer in male rats. Exp. Anim..

[204] Guo J., Guo J., Cheng B., Gong M., Sun X., Zhang H., Ma J. (2024). Ozone enhances the efficacy of radiation therapy in esophageal cancer. J. Radiat. Res..

[205] Dogan R., Hafız A.M., Kiziltan H.S., Yenigun A., Buyukpinarbaslili N., Eris A.H., Ozturan O. (2018). Effectiveness of radiotherapy+ozone on tumoral tissue and survival in tongue cancer rat model. Auris Nasus Larynx.

[206] Kızıltan H.Ş., Bayir A.G., Yucesan G., Eris A.H., İdin K., Karatoprak C., Aydin T., Akcakaya A., Mayadagli A. (2015). Medical ozone and radiotherapy in a peritoneal, Erlich-ascites, tumor-cell model. Altern. Ther. Health Med..

[207] Karagulle O.O., Yurttas A.G. (2022). Ozone combined with doxorubicin exerts cytotoxic and anticancer effects on Luminal-A subtype human breast cancer cell line. Rev. Assoc. Med. Bras..

[208] Fahmy S.A., Ramzy A., Sawy A.M., Nabil M., Gad M.Z., El-Shazly M., Aboul-Soud M.A.M., Azzazy H.M.E. (2022). Ozonated Olive Oil: Enhanced Cutaneous Delivery via Niosomal Nanovesicles for Melanoma Treatment. Antioxidants.

[209] Zhang Y., Zhang C., Wu B., Li C., Lin J., Huang P. (2023). Thermoresponsive Ozone-Enriched Spray Gel for Postsurgical Treatment of Hepatocellular Carcinoma. ACS Nano.

[210] Lu J., Zheng R., Shi Z., Gao X., Li Y., Fahad A., Ufurahi-Pambe N., Jin Z., Chen S., Xie W. (2024). Intracellular Criegee’s mechanism-based synergistic ozone therapy mediated by oleogels for cancer treatment. J. Control Release.

[211] Kang K.A., Hyun J.W. (2017). Oxidative Stress, Nrf2, and Epigenetic Modification Contribute to Anticancer Drug Resistance. Toxicol. Res..

[212] Karagülle O.O., Yurttaş A.G. (2023). Synergistic effects of ozone with doxorubicin on the proliferation, apoptosis and metastatic profile of luminal-B type human breast cancer cell line. Tissue Cell.

[213] Costanzo M., Romeo A., Cisterna B., Calderan L., Bernardi P., Covi V., Tabaracci G., Malatesta M. (2020). Ozone at low concentrations does not affect motility and proliferation of cancer cells in vitro. Eur. J. Histochem..

[214] Inguscio C.R., Carton F., Cisterna B., Rizzi M., Boccafoschi F., Tabaracci G., Malatesta M. (2024). Low ozone concentrations do not exert cytoprotective effects on tamoxifen-treated breast cancer cells in vitro. Eur. J. Histochem..

[215] Knezevic N.N., Jovanovic F., Voronov D., Candido K.D. (2018). Do Corticosteroids Still Have a Place in the Treatment of Chronic Pain?. Front. Pharmacol..

[216] Williams D.M. (2018). Clinical Pharmacology of Corticosteroids. Respir. Care.

[217] Dixit A., Agarwal B., Singh K., Chand P., Rao J., Mishra N. (2024). Comparative Evaluation of Normal Saline Gel and Ozone Therapy on Soft and Hard Tissue Health in Dental Implant Surgery. Indian J. Dent. Res..

[218] Yang C., Chen Q. (2023). Effects of ozone combined with articular injection of sodium hyaluronate on patients with knee osteoarthritis and their inflammatory factors and hemorheological indices. Afr. Health Sci..

[219] Foula A.S., Sabry L.S., Elmulla A.F., Kamel M.A., Hozien A.I. (2023). Ultrasound-guided Shoulder Intraarticular Ozone Injection Versus Pulsed Radiofrequency Application for Shoulder Adhesive Capsulitis: A Randomized Controlled Trial. Pain Physician.

[220] Izadi M., Jafari-Oori M., Eftekhari Z., Jafari N.J., Maybodi M.K., Heydari S., Vahedian-Azimi A., Atkin S.L., Jamialahmadi T., Sahebkar A. (2024). Effect of Ozone Therapy on Diabetes-related Foot Ulcer Outcomes: A Systematic Review and Meta-analysis. Curr. Pharm. Des..

[221] Torul D., Omezli M.M., Avci T. (2023). Investigation of the clinical efficacy of CGF and ozone in the management of alveolar osteitis: A randomized controlled trial. Clin. Oral Investig..

